# Supplementary material for: Design and Synthesis of π-Extended Resveratrol Analogues and In Vitro Antioxidant and Anti-Inflammatory Activity Evaluation
Source: Molecules. 2021 Jan 26;26(3):646. doi: 10.3390/molecules26030646 (PMC7865754; doi:10.3390/molecules26030646)
Supplement: Supplementary file 1 [file molecules-26-00646-s001.pdf]

Supplementary Materials

Design and Synthesis of π-extended resveratrol analogues and *in vitro* antioxidant and anti-inflammatory activity evaluation

Ki Yoon Nam,[1] Kongara Damodar,[1] Yeontaek Lee,[2] Lee Sul Park,[1] Ji Geun Gim,[1] Jae Phil Park,[2] Seong Ho Jeon*[,2] and Jeong Tae Lee*[,1]

[1]*Department of Chemistry and Institute of Applied Chemistry, Hallym University, Chuncheon 24252, Republic of Korea*

[2]*Department of Life Science and Multidisciplinary Genome Institute, Hallym University, Chuncheon 24252, Republic of Korea*

[*]Corresponding author. Tel.: +82 33 248 2071 (J.T.L.); +82 33 248 2096 (S.H.J.)

E-mail address: jtshl@hallym.ac.kr (J.T.L.); leo900516@gmail.com (J.T.L.); sjeon@hallym.ac.kr (S.H.J.)

Table of Contents

General remarks……………………………………………………………...……….2

Experimental procedure & Product Characterization……………………………………2

Antioxidant assay using ABTS……………………………………………………..22

NO assay and cell viability assay…………………......…………………..……..…………22

Statistical Analysis…………………………………………………………………..22

Reverse transcriptase-polymerase chain reaction (RT-PCR)…………………………….24

NMR spectra………………………………………………………………...……25

IR spectra……………………………………………………………………………31

UV-Vis spectra………………………………………………………………………33

General remarks

All starting materials, chemical and biological reagents, and solvents were purchased from commercial sources and used without any additional purification. Solvents used for reactions were purchased as either anhydrous grade products or freshly distilled using proper dehydrating agents. ^{1}H NMR and ^{13}C NMR spectra were recorded at room temperature on a Varian Mercury TM 300 MHz FT-NMR and JNM-ECZ400S (400 MHz) FT-NMR (JEOL Ltd., Tokyo, Japan). Chloroform-*d*/acetone-d_6/methanol-d_4/dimethyl sulfoxide-d_6 was used as a solvent. Chemical shifts (δ) were reported in parts per million (ppm) downfield relative to tetramethylsilane (TMS). The following pattern was used for ^{1}H NMR data: δ value (multiplicity (singlet = s), (doublet = d), (triplet = t), (quartet = q), (doublet of doublet = dd), and (multiplet = m), number of protons, coupling constants (J) quoted in Hz). Mass spectra analyses were taken on a JMS-700 (JEOL Ltd) spectrometer at the central laboratory of Kangwon National University. Melting points were uncorrected and observed on a MEL-TEMP II (Triad Scientific, Manasquan, NJ, USA) open capillary melting points apparatus. All reactions were monitored through thin-layer chromatography utilizing Merck silica gel plates 60 F_{254} and spots visualization was accomplished by UV light absorption and/or p-anisaldehyde/phosphomolybdic acid stain. Silica gel 60 [230–400 mesh (40–63 μm), Merck, Darmstadt, Germany] was employed for chromatographic purification. Antioxidant activity was tested in the dark room by ABTS assay. UV-1800 (Shimadzu Corporation, Japan) was used to measure UV absorption. Hellma's 104.600-QC Quartz cell was used as a UV cell. IC_{50} values were obtained using OriginPro 8.0 software.

Experimental procedure & Product Characterization

3,5-Dibenzyloxybenzaldehyde (9)

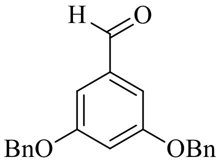

9

R_f = 0.50 (EtOAc/hexane = 1/4); ^{1}H NMR (300 MHz, $CDCl_3$): δ 9.85 (1H, s), 7.39–7.31 (10H, m), 7.08 (2H, s), 6.84 (1H, s), 5.06 (4H, s); ^{13}C NMR (75 MHz, $CDCl_3$): δ 191.3, 160.4,

138.5, 136.3, 128.6, 128.1, 127.4, 108.8, 108.4, 70.5.

Diethyl 3,5-bis(benzyloxy)benzylphosphonate (10)

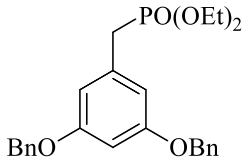

10

R_f = 0.3 (EtOAc/hexane = 1/1); ^{1}H NMR (300 MHz, $CDCl_3$): δ 7.39–7.27 (10H, m), 6.54 (2H, d, *J* = 2.4 Hz), 6.49 (1H, t, *J* = 2.4 Hz) 4.99 (4H, s), 4.01–3.93 (4H, m), 3.06 (2H, d, *J* = 21.5 Hz), 1.23 (6H, t, *J* = 7.1 Hz).

Benzyl 6-(benzyloxy)-2-naphthoate (12)

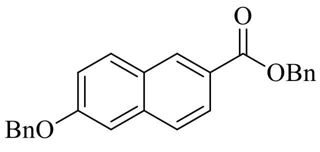

12

R_f = 0.64 (EtOAc/hexane = 1/2); ^{1}H NMR (300 MHz, $CDCl_3$): δ 8.52 (1H, s), 8.02 (1H, d, *J* = 8.4 Hz), 7.80 (1H, d, *J* = 9.0 Hz), 7.69 (1H, d, *J* = 8.4 Hz), 7.44–7.19 (12H, m), 5.38 (2H, s), 5.14 (2H, s); ^{13}C NMR (75 MHz, $CDCl_3$): δ 166.5, 158.6, 137.1, 136.5, 136.3, 130.9, 128.5, 128.1, 127.4, 126.8, 125.9, 125.4, 119.8, 107.3, 70.2, 66.7

6-Benzyloxy-2-naphthaldehyde (13)

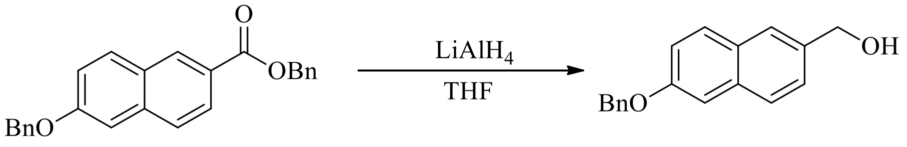

12

R_f = 0.32 (EtOAc/hexane = 1/7); ^{1}H NMR (300 MHz, DMSO-d_6): δ 7.79–7.71 (3H, m), 7.50 (1H, s), 7.47 (1H, s), 7.41–7.31 (5H, s), 7.19 (1H, dd, *J* = 8.8, 2.9 Hz), 5.20 (2H, s), 5.14 (1H, br s), 4.61 (2H, d, *J* = 5.4 Hz); ^{13}C NMR (75 MHz, DMSO-d_6): δ 155.6, 137.5, 136.7, 132.9, 128.8, 128.1, 128.0, 127.4, 127.3, 126.1, 125.5, 124.1, 118.3, 107.2, 69.2, 62.8.

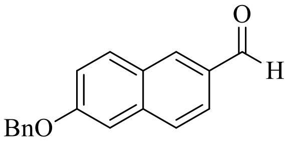

13

The reaction mixture of (6-(benzyloxy)naphthalen-2-yl)methanol (0.36 g, 1.36 mmol) and IBX (2-iodoxybenzoic acid, 0.57 g, 2.04 mmol) in DMSO (dimethyl sulfoxide, 5 mL) was stirred for 30 minutes at room temperature under argon. Then ethyl acetate (15 mL) was added to the reaction mixture and the organic layer was washed with sat. $NaHCO_3$ (20 mL x 3) and brine (20 mL x 3). The organic layer was dried over Na_2SO_4 and solvent was removed *in vacuo*. The desired product 6-benzyloxy-2-naphthaldehyde **1.2** was obtained as a light brown solid (0.36 g, 94.1 %). R_f = 0.31 (EtOAc/hexane = 1/7); ^{1}H NMR (300 MHz, $CDCl_3$): δ 10.07 (1H, s), 8.25 (1H, s), 7.90 (2H, d, *J* = 8.7 Hz), 7.79 (1H, d, *J* = 8.7 Hz), 7.24–7.49 (7H, m), 5.21 (2H, s); ^{13}C NMR (75 MHz, $CDCl_3$): δ 191.3, 159.2, 138.0, 136.3, 133.6, 132.5, 131.0, 128.5, 128.1, 128.0, 127.6, 127.3, 123.6, 120.0, 107.7, 70.3; MS (EI^+) *m/z*(%): 262 ($[M]^+$, 100).

Synthesis of *N*-(3-(benzyloxy)phenyl)acetamide (14)

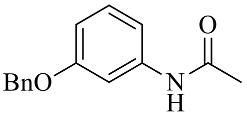

14

To a stirred solution of *N*-(3-hydroxyphenyl)acetamide (5.0 g, 33.08 mmol) in acetone (130 mL) was added K_2CO_3 (18.23 g, 132.31 mmol) under argon atmosphere and stirred for 30 min at room temperature. Benzyl bromide (3.93 mL, 33.08 mmol) was added dropwise and stirred for 2 h. After completion of the reaction, the solid was filtered off and the filtrate was concentrated under reduced pressure. The residue was dissolved in EtOAc (70 mL), washed with 2N HCl (2 x 15 mL), H_2O (2 x 20 mL) and brine (20 mL), dried over anhyd. Na_2SO_4 and concentrated *in vacuo*. The crude was purified by column chromatography ($MeOH/CH_2Cl_2$ = 1:19) afford the desired product **14** (7.82 g, 98.0 %) as white solid. R_f = 0.3 ($MeOH/CH_2Cl_2$ = 1/19); ^{1}H NMR (300 MHz, $CDCl_3$) δ 7.61 (1H, s), 7.39–7.23 (6H, m), 7.15 (1H, t, *J* = 7.3 Hz), 6.97 (1H, d, *J* = 7.8 Hz), 6.69 (1H, dd, *J* = 8.1, 2.2 Hz), 5.00 (2H, s), 2.12 (3H, s); ^{13}C NMR (75 MHz, $CDCl_3$) δ 168.4, 159.1, 139.0, 136.7, 129.5, 128.4, 127.8, 127.4, 112.2, 110.8, 106.4, 69.9, 24.7; EI-MS *m/z* 241 ($[M]^+$, 100%).

Synthesis of 7-(benzyloxy)-2-chloroquinoline-3-carbaldehyde (15)

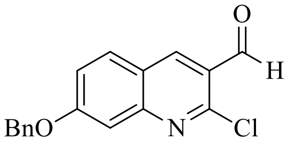

15

To a stirred solution of compound **14** (2.0 g, 8.20 mmol) in anhydrous *N*, *N*-dimethylformamide (1.93 mL, 24.87 mmol) was added $POCl_3$ (9.38 mL, 100.3 mmol) dropwise under argon atmosphere at 0 ºC. The reaction mixture was warmed to 90 ºC and stirred for 2 h. After completion of the reaction, cooled to room temperature and ice cold water was added slowly and stirred until a brown solid is formed. The solid was filtered, washed with H_2O and dried to afford the title compound **15** (1.91 g, 77.5 %). R_f = 0.74 (EtOAc/hexane = 1/1); ^{1}H NMR (300 MHz, $CDCl_3$) δ 10.45 (1H, s), 8.60 (1H, s) 7.81 (1H, d, *J* = 9.0 Hz), 7.46–7.29 (7H, m), 5.20 (2H, s); ^{13}C NMR (75 MHz, $CDCl_3$) δ 188.6, 163.1, 151.7, 150.9, 139.3, 135.5, 130.7, 128.6, 128.3, 127.5, 124.5, 121.8, 121.6, 108.1, 70.7; EI-MS *m/z* 297 ($[M]^+$, 100%).

Synthesis of 7-(benzyloxy)quinoline-3-carbaldehyde (16)

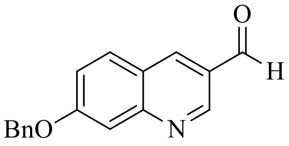

16

To a stirred solution of compound **15** (1.0 g, 3.36 mmol) in degassed *N*, *N*-dimethylformamide (6 mL) was added $Pd(PPh_3)_4$ (0.19 g, 0.17 mmol, 0.05 equiv.) and triethylamine (7.50 mL, 53.74 mmol) under argon atmosphere at room temperature. To this mixture, formic acid (0.8 mL, 18.14 mmol) was added dropwise at 0 °C and degassed for 4 min. The reaction mixture was warmed to 110 ºC and stirred for 2 h. After completion of the reaction, cooled to room temperature and extracted with EtOAc (3 x 25 mL). The combined organic layer was washed with H_2O (3 x 15 mL) and brine (15 mL), dried over

anhyd. Na_2SO_4 and concentrated *in vacuo*. The crude was purified by column chromatography (EtOAc/hexane = 1/2) to afford the desired product **16** (0.66 g, 74.2 %) as pale yellow solid. R_f = 0.26 (EtOAc/hexane = 1/2); ^1H NMR (300 MHz, $CDCl_3$) δ 10.16 (1H, s), 9.24 (1H, d, *J* = 2.1 Hz), 8.51 (1H, d, *J* = 2.1 Hz), 7.85 (1H, d, *J* = 9.0 Hz), 7.55 (1H, d, *J* = 2.4 Hz), 7.48–7.33 (6H, m), 5.24 (2H, s); ^{13}C NMR (75 MHz, $CDCl_3$) δ 190.2, 162.3, 152.7, 150.0, 138.8, 135.8, 130.5, 128.7, 128.3, 127.6, 127.2, 122.3, 121.5, 109.1, 70.6; EI-MS *m/z* 263 ($[M]^+$, 100%).

6-(Benzyloxy)-2-methylquinoline (18)

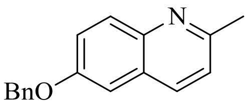

18

To a stirred solution of 4-(benzyloxy)aniline **17** (0.83 g, 4.16 mmol) and ethyl vinyl ether (0.88 mL, 9.15 mmol) in anhyd. toluene (6 mL) was added iodine (0.053 g, 0.21 mmol) under argon atmosphere at room temperature. The reaction mixture was warmed to 80 ºC and stirred for 2 h. After completion of the reaction, cooled to room temperature and aqueous saturated $Na_2S_2O_3$ solution was added to it. Two layers separated and the aqueous layer was extracted with EtOAc (3 x 25 mL). The combined organic layer was washed with H_2O (3 x 20 mL) and brine (20 mL), dried over anhyd. Na_2SO_4 and concentrated *in vacuo*. The crude was purified by column chromatography (EtOAc/hexane = 1/4) to afford the desired product **18** (0.51 g, 49.1 %) as beige solid. R_f = 0.5 (EtOAc/hexane = 1/1); ^1H NMR (300 MHz, $CDCl_3$) δ 7.91 (1H, d, *J* = 9.0 Hz), 7.88 (1H, d, *J* = 8.4 Hz), 7.31–7.46 (6H, m), 7.19 (1H, d, *J* = 8.4 Hz), 7.09 (1H, d, *J* = 2.7 Hz), 5.14 (2H, s), 2.69 (3H, s); ^{13}C NMR (75 MHz, $CDCl_3$) δ 156.4, 156.3, 144.1, 136.7, 134.9, 130.2, 128.5, 127.9, 127.4, 127.2, 122.1, 122.0, 107.0, 70.5, 25.1; EI-MS *m/z* 250 ($[M+H]^+$, 130.

5-(Benzyloxy)-2-bromobenzaldehyde (20)

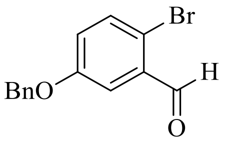

20

To a stirred solution of compound **19** (5 g, 24.87 mmol) in anhyd. *N*, *N*-dimethylformamide (30 mL) was added K_2CO_3 (5.16 g, 37.30 mmol) under argon atmosphere and stirred for 30 min at room temperature. Benzyl bromide (3.25 mL, 27.36 mmol) was added dropwise and the mixture was stirred for 2 h. After completion of the reaction, neutralized with 3N HCl and extract with EtOAc (3 x 40 mL). The combined organic layer was washed with H_2O (3 x 25 mL) and brine (25 mL), dried over Na_2SO_4 and concentrated *in vacuo*. The crude was purified by column chromatography (EtOAc/hexane = 1/16) to afford the desired product **20** (6.52 g, 90.1 %) as white solid. R_f = 0.85 (EtOAc/hexane = 1/4); ^{1}H NMR (300 MHz, $CDCl_3$) δ 10.26 (1H, s), 7.50–7.28 (7H, m), 7.06 (1H, dd, *J* = 8.7, 3.0 Hz), 5.06 (2H, s); ^{13}C NMR (75 MHz, $CDCl_3$) δ 191.2, 158.4, 135.9, 134.5, 134.1, 128.6, 128.2, 127.4, 123.5, 117.9, 114.2, 70.6; EI-MS *m/z* 292 ($[M]^+$+2, base), 290 ($[M]^+$), 212, 199.

Methyl 7-(benzyloxy)isoquinoline-3-carboxylate (21)

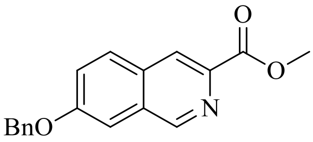

21

To a stirred solution of compound **20** (1.0 g, 3.36 mmol) in anhyd. DMSO (5 mL) was added methyl 2-acetamidoacrylate (0.58 g, 4.02 mmol), triethylamine (1.51 mL, 10.82 mmol), tri (*o*-tolyl) phosphine (0.19 g, 0.62 mmol) and $Pd(OAc)_2$ (0.06 g, 0.30 mmol) and degassed for 5-10 min. The reaction mixture was warmed to 110 ºC and stirred for 18 h. After completion of the reaction, cooled to room temperature, aqueous saturated NH_4Cl solution (5 mL) was added and extracted with EtOAc (3 x 25 mL). The combined organic layer was washed with H_2O (3 x 20 mL) and brine (20 mL), dried over anhyd. Na_2SO_4 and concentrated *in vacuo*. The crude was purified by column chromatography (EtOAc/hexane = 1/2) to afford the desired product **21** (0.66 g, 74.2 %) as pale brown solid. R_f = 0.3 (EtOAc/hexane = 1/1); ^{1}H NMR (300 MHz, $CDCl_3$) δ 9.14 (1H, s), 8.44 (1H, s), 7.79 (1H, d, *J* = 9.0 Hz), 7.44–7.30 (7H, m), 5.17 (2H, s), 4.00 (3H, s); ^{13}C NMR (75 MHz, $CDCl_3$) δ 165.9, 159.1, 150.7, 139.7,

135.7, 131.2, 130.6, 129.3, 128.4, 128.0, 127.2, 124.1, 123.4, 106.3, 70.2, 52.4; EI-MS *m/z* 293 ($[M]^+$, base), 262, 234.

(7-(Benzyloxy)isoquinolin-3-yl)methanol (22)

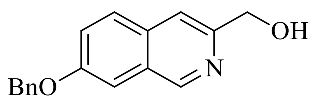

22

To a stirred solution of compound **21** (0.89 g, 3.05 mmol) in anhydrous THF (8 mL) was added LiAlH4 solution (1.0 M in THF, 3.96 mL, 3.96 mmol) dropwise at 0 °C under argon atmosphere. The reaction was warmed to room temperature and stirred for 1 h. After completion of the reaction, the reaction mixture was cooled to 0 °C and the excess LiAlH4 was quenched by the slow addition of aqueous saturated Na_2CO_3 solution (4 mL) and stirred for 20 min. The resulting suspension was filtered, washed with EtOAc (50 mL). The filtrate was washed with H_2O (3 x 15 mL) and brine (15 mL), dried over anhydrous Na_2SO_4 and concentrated under reduced pressure. The crude was purified by column chromatography ($MeOH/CH_2Cl_2$ = 1/19) to afford the pure product **22** (0.49 g, 60.5%) as pale brown liquid. R_f = 0.1 (EtOAc/hexane = 1/1); ^1H NMR (300 MHz, $CDCl_3$) δ 8.99 (1H, s), 7.63 (1H, d, *J* = 9.0 Hz), 7.58 (1H, s), 7.47–7.32 (6H, m), 7.18 (1H, s), 5.12 (2H, s), 5.08 (1H, br s), 4.91 (2H, s); ^{13}C NMR (75 MHz, $CDCl_3$) δ 157.2, 151.5, 150.2, 136.2, 132.0, 128.7, 128.5, 128.0, 127.9, 127.3, 123.9, 116.6, 106.2, 70.2, 64.9; EI-MS *m/z* 265 ($[M]^+$, base), 263.

7-(Benzyloxy)isoquinoline-3-carbaldehyde (23)

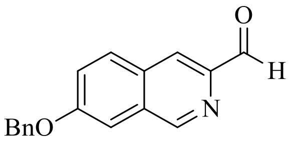

23

To a stirred solution of IBX (0.08 g, 0.28 mmol) in anhydrous DMSO (2.5 mL) was added compound **22** (0.05 g, 0.19 mmol) in anhydrous DMSO (2.5 mL) under argon atmosphere at room temperature and he reaction mixture was stirred for 1 h. After completion of the reaction, diluted with EtOAc (25 mL) and washed with aqueous saturated $NaHCO_3$ solution (3 x 10 mL) and brine (10 mL). The organic layer was dried over anhydrous Na_2SO_4 and concentrated under reduced pressure. The crude was purified by column chromatography (EtOAc/hexane = 1/2) to afford the pure product **23** (0.03 g, 66.0 %) as pale brown solid. R_f = 0.6 (EtOAc/hexane = 1/1); ^{1}H NMR (300 MHz, $CDCl_3$) δ 10.19 (1H, s), 9.21 (1H, s), 8.29 (1H, s), 7.90 (1H, d, *J* = 8.7 Hz), 7.51–7.35 (7H, m), 5.23 (2H, s); ^{13}C NMR (75 MHz, $CDCl_3$) δ 192.8, 159.8, 151.4, 145.9, 135.8, 132.2, 130.7, 130.2, 128.7, 128.3, 127.4, 124.6, 121.2, 106.7, 70.6; EI-MS *m/z* 263 ($[M]^+$, base).

Ethyl 2-((4-fluoro-2-nitrophenyl)amino)propanoate (25)

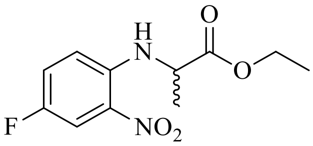

25

To a stirred solution of compound **24** (0.34 mL, 3.14 mmol) in pyridine (6 mL) were added L-alanine ethyl ester hydrochloride (0.72 g, 4.71 mmol) and triethylamine (0.88 mL, 6.29 mmol) under argon atmosphere and the reaction mixture was stirred at 80 ° C for 17 h. After completion of the reaction, cooled to room temperature, diluted with EtOAc (40 mL) and sequentially washed with 10% aqueous $CuSO_4$ (10 mL) solution, H_2O (3 x 10 mL) and brine

(10 mL). The organic layer was dried over anhyd. Na_2SO_4 and concentrated *in vacuo*. The crude was purified by column chromatography (EtOAc/hexane = 1/10) to afford the desired product **25** (0.75 g, 92.6%) as orange solid. R_f = 0.4 (EtOAc/hexane = 1/8); ^{1}H NMR (300 MHz, $CDCl_3$) δ 8.16 (1H, d, *J* = 6.8 Hz), 7.87 (1H, dd, *J* = 9.0, 3.1 Hz), 7.26–7.19 (1H, m), 6.70 (1H, dd, *J* = 9.0, 4.4 Hz), 4.30–4.19 (3H, m), 1.60 (3H, d, *J* = 6.8 Hz), 1.29 (3H, t, *J* = 7.2 Hz); ^{13}C NMR (75 MHz, $CDCl_3$) δ 172.1, 152.8 (*J* = 237.4 Hz), 140.8, 131.9, 124.3 (*J* = 23.3 Hz), 115.1 (*J* = 7.1 Hz), 112.3 (*J* = 26.2 Hz), 61.6, 51.8, 18.6, 14.1.

7-Fluoro-3-methylquinoxalin-2(1*H*)-one (26)

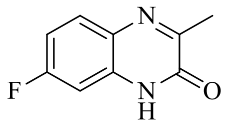

26

To a stirred solution of compound **25** (0.75 g, 2.93 mmol) in EtOH/H_2O (10/1; 8.8 mL) was added tin (II) chloride dihydrate (2.64 g, 11.71 mmol) at room temperature and the reaction was refluxed for 1 h. After completion of the reaction, cooled to room temperature, basified using 2N NaOH, filtered through Celite® pad and washed with diethyl ether (10 mL) and tetrahydrofuran (5 mL). The filtrate was concentrated under reduced pressure. The crude was dissolved in CH_3CN (8 mL), 2,3-dichloro-5,6-dicyano-1,4-benzoquinone (1.0 g, 4.39 mmol) was added to it and stirred for 30 min. After completion of the reaction, filtered through Celite® pad and washed with EtOAc/THF (4/1; 20 mL). The filtrate was diluted with EtOAc (25 mL), sequentially washed with aqueous saturated $NaHCO_3$ (2 x 15 mL), H_2O (2 x 15 mL) and brine (15 mL), dried over anhyd. Na_2SO_4 and concentrated under reduced pressure. The crude was triturated with hexane and diethyl ether to give the desired product **26** (0.52 g, quantitative) as brown solid. R_f = 0.4 (EtOAc/hexane = 1/1); ^{1}H NMR (300 MHz, DMSO-d_6)

δ 12.32 (1H, br s), 7.72 (1H, dd, *J* = 8.7, 6.0 Hz), 7.10 (1H, td, *J* = 8.7, 2.6 Hz), 6.99 (1H, dd, *J* = 9.8, 2.6 Hz), 2.38 (3H, s); ^{13}C NMR (75 MHz, DMSO-d_6) δ 161.3 (*J* = 237.4 Hz), 157.8, 154.3, 133.0 (*J* = 12.5 Hz), 129.7 (*J* = 10.6 Hz), 128.4, 110.4 (*J* = 23.7 Hz), 100.8 (*J* = 26.5 Hz), 20.0.

3-Chloro-6-fluoro-2-methylquinoxaline (27)

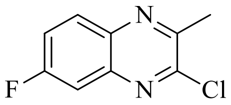

27

To a stirred solution of compound **26** (2.37 g, 13.30 mmol) in *N*, *N*-dimethylaniline (20 mL) was added phosphoryl chloride (37.31 mL, 399.1 mmol) dropwise at room temperature under argon atmosphere. The reaction was warmed to 110 ºC and stirred for 1 h. After completion of the reaction, cooled to room temperature and H_2O (5 mL) was added dropwise. The reaction mixture was extracted with EtOAc (3 x 30 mL). The combined organic layer was washed with H_2O (3 x 20 mL) and brine (20 mL), dried over anhyd. Na_2SO_4 and concentrated under reduced pressure. The crude was purified by column chromatography (EtOAc/hexane = 1/16) to afford the desired product **27** (2.26 g, 86.1%) as pale yellow solid. R_f = 0.5 (EtOAc/hexane = 1/8); ^{1}H NMR (300 MHz, $CDCl_3$) δ 7.98 (1H, dd, *J* = 9.1, 5.8 Hz), 7.58–7.45 (2H, m), 2.81 (3H, s); ^{13}C NMR (75 MHz, $CDCl_3$) δ 162.6 (*J* = 244.6 Hz), 151.9 (*J* = 3.1 Hz), 148.8, 141.6 (*J* = 13.1 Hz), 138.1, 130.4 (*J* = 10.0 Hz), 120.0 (*J* = 25.6 Hz), 111.9 (*J* = 22.2 Hz), 23.1.

6-Fluoro-2-methylquinoxaline (28)

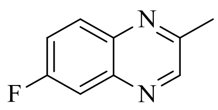

28

To a stirred solution of compound **27** (1.18 g, 6.02 mmol) in degassed *N*, *N*-dimethylformamide (15 mL) was added $Pd(PPh_3)_4$ (0.35 g, 0.30 mmol) and triethylamine (13.43 mL, 96.27 mmol) under argon atmosphere at room temperature. To this mixture, formic acid (1.23 mL, 32.49 mmol) was added dropwise at 0 °C and degassed for 4 min. The reaction mixture was warmed to 110 ºC and stirred for 2 h. After completion of the reaction, cooled to room temperature and extracted with EtOAc (3 x 30 mL). The combined organic layer was washed with H_2O (3 x 25 mL) and brine (25 mL), dried over anhyd. Na_2SO_4 and concentrated *in vacuo*. The crude was purified by column chromatography (EtOAc/hexane = 1/8) to afford the desired product **28** (0.81 g, 5.00 mmol, 83.1 %) as beige solid. R_f = 0.4 (EtOAc/hexane = 1/4); ^{1}H NMR (300 MHz, $CDCl_3$) δ 8.71 (1H, s), 7.98 (1H, dd, *J* = 9.0, 5.8 Hz), 7.66 (1H, dd, *J* = 9.0, 3.0 Hz) 7.53–7.46 (1H, m), 2.75 (3H, s); ^{13}C NMR (75 MHz, $CDCl_3$) δ 161.9 (*J* = 248.8 Hz), 152.9, 146.5, 141.6 (*J* = 12.8 Hz), 139.2, 130.6 (*J* = 9.7 Hz), 120.0 (*J* = 25.6 Hz), 112.7 (*J* = 21.3 Hz), 22.4.

6-(Benzyloxy)-2-methylquinoxaline (29)

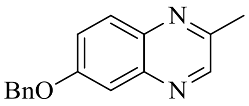

29

To a stirred suspension of NaH (60% in mineral oil, 0.40 g, 9.95 mmol) in *N*, *N*-dimethylformamide (4 mL) was added benzyl alcohol (0.67 mL, 6.47 mmol) in *N*, *N*-dimethylformamide (3 mL) dropwise under argon atmosphere at room temperature and stirred for 30 min. Compound **28** (0.80 g, 4.98 mmol) in *N*, *N*-dimethylformamide (8 mL)

was added dropwise to the above mixture. The resulting mixture was warmed 100 ºC and stirred for 2h. After completion of the reaction, cooled to 0 ºC, quenched with H_2O (5 mL) and extracted with EtOAc (3 x 25 mL). The combined organic layer was washed with H_2O (3 x 20 mL) and brine (20 mL), dried over anhyd. Na_2SO_4 and concentrated *in vacuo*. The crude was purified by column chromatography (EtOAc/hexane = 1/4) to afford the desired product **29** (1.04 g, 83.4 %) as pale yellow solid. (R_f = 0.4 (EtOAc/hexane = 1/1); ^{1}H NMR (300 MHz, $CDCl_3$) δ 8.63 (1H, s), 7.89 (1H, d, *J* = 9.0 Hz), 7.48–7.30 (7H, m), 5.19 (2H, s), 2.72 (3H, s); ^{13}C NMR (75 MHz, $CDCl_3$) δ 159.0, 151.1, 145.7, 142.3, 138.3, 136.2, 129.7, 128.6, 128.1, 127.5, 123.2, 108.3, 70.6, 22.2; EI-MS *m/z* 250 ($[M]^+$, base).

6-(Benzyloxy)-2-methylquinazoline (30)

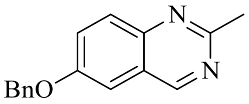

30

To a stirred solution of compound **20** (5 g, 17.17 mmol) in anhyd. *N*, *N*-dimethylformamide (50 mL) were added acetamidine hydrochloride (1.79 g, 18.89 mmol), L-proline (0.40 g, 3.43 mmol), Cs_2CO_3 (16.79 g, 51.52 mmol) and CuI (0.33 g, 1.72 mmol) under argon atmosphere at room temperature. The mixture was warmed to 110 ºC and stirred for 18 h. After completion of the reaction, cooled to room temperature, filtered through Celite® pad and washed with EtOAc (20 mL). The filtrate was neutralized with 1N HCl and extract with EtOAc (3 x 50 mL). The combined organic layer was washed with H_2O (3 x 30 mL) and brine (30 mL), dried over Na_2SO_4 and concentrated *in vacuo*. The crude was purified by column chromatography (EtOAc/hexane = 1/2) to afford the desired product **30** (1.16 g, 27.0 %) as brown solid. R_f = 0.3 (EtOAc/hexane = 1/2); ^{1}H NMR (300 MHz, $CDCl_3$) δ 9.16 (1H, s), 7.84 (1H, d, *J* = 9.3 Hz) 7.57 (1H, dd, *J* = 9.3, 2.7 Hz), 7.46–7.33 (5H, m), 7.16 (1H, d, *J* = 2.4 Hz), 5.15 (2H, s), 2.85 (3H, s); ^{13}C NMR (75 MHz, $CDCl_3$) δ 162.4, 158.5, 156.8, 146.5, 136.0, 129.2, 128.6, 128.1, 127.3, 127.1, 123.5, 105.3, 70.5, 26.1; EI-MS *m/z* 250 ($[M]^+$, base).

(*E*)-2-Benzyloxy-6-(3,5-bis(benzyloxy)styryl)naphthalene (31)

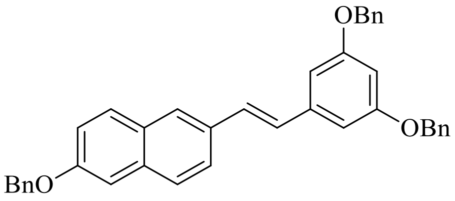

31

To a solution of the compound **10** (0.4 g, 0.9 mmol) in anhydrous DMF (5 mL) was added sodium hydride (60% NaH in mineral oil, 0.11 g, 2.72 mmol) at 0 ºC under argon. After 30 minutes, compound **13** (0.24 g, 0.9 mmol) was added and the reaction mixture was stirred for 2 hours at room temperature. The reaction was quenched by 1N HCl at 0 ºC (pH ≈ 5). After filtration with Celite® 545, the organic layer was extracted with DCM (15 mL) and washed with brine (20 mL x 3). The solvent was evaporated *in vacuo* to give the crude product, which was purified on a silica gel (DCM:hexanes = 1:1) to afford the desired product **31** (0.43 g, 86.0 %) as a white solid. R_f = 0.46 (EtOAc/hexane = 1/6); ^{1}H NMR (300 MHz, $CDCl_3$): δ 7.68–7.77 (4H, m), 7.32–7.50 (15H, m), 7.17–7.28 (2H, m), 7.11 (1H, s), 7.06 (1H, s), 6.81 (2H, d, *J* = 2.1 Hz), 6.55 (1H, t, *J* = 2.2 Hz), 5.17 (2H, s), 5.08 (4H, s); ^{13}C NMR (75 MHz, $CDCl_3$): δ 160.1, 156.9, 139.6, 136.9, 136.8, 134.2, 132.6, 129.5, 129.4, 129.2, 128.5, 127.9, 127.4, 127.2, 126.5, 124.1, 119.3, 107.4, 105.9, 101.6, 70.2; MS (EI$^+$) *m/z*(%): 548 ([M]$^+$, 100).

Synthesis of (*E*)-7-(benzyloxy)-3-(3,5-bis(benzyloxy)styryl)quinoline (32)

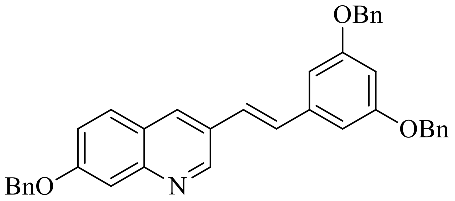

32

To a stirred suspension of NaH (60% in mineral oil, 0.27 g, 6.73 mmol) in *N*, *N*-dimethylformamide (3 mL) was added compound **10** (1.09 g, 2.47 mmol) in *N*, *N*-dimethylformamide (3 mL) dropwise under argon atmosphere at room temperature and stirred for 30 min. Compound **4** (0.60 g, 2.24 mmol) in *N*, *N*-dimethylformamide (3 mL) was added dropwise to the above mixture and the reaction mixture stirred for 2h. After completion of the reaction, cooled to 0 ºC, quenched with H_2O (3 mL) and extracted with EtOAc (3 x 25 mL). The combined organic layer was washed with H_2O (3 x 20 mL) and brine (20 mL),

dried over anhyd. Na_2SO_4 and concentrated *in vacuo*. The crude was purified by column chromatography ($MeOH/CH_2Cl_2$ = 1/19) to afford the desired product **5** (1.10 g, 89.4 %) as pale yellow solid. R_f = 0.41 (EtOAc/hexane = 1/2); ^1H NMR (300 MHz, $CDCl_3$) δ 8.97 (1H, s), 8.05 (1H, s) 7.68 (1H, d, J = 9.0 Hz), 7.48–7.22 (17H, m), 7.13 (2H, s), 6.79 (2H, s), 6.56 (1H, s), 5.19 (2H, s), 5.06 (4H, s); ^{13}C NMR (75 MHz, $CDCl_3$) δ 160.3, 159.8, 149.7, 149.2, 139.1, 137.0, 136.5, 131.9, 129.8, 128.8, 128.6, 128.5, 128.4, 128.0, 127.9, 127.5, 127.4, 126.1, 123.4, 120.4, 109.1, 106.3, 102.4, 70.4; EI-MS *m/z* 549 ($[M]^+$, base), 458, 368.

(*E*)-7-(Benzyloxy)-3-(3,5-bis(benzyloxy)styryl)isoquinoline (33)

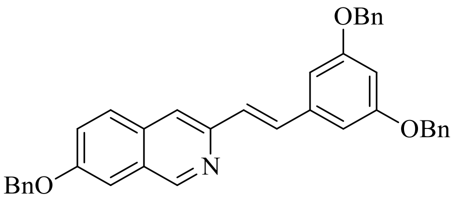

33

To a stirred suspension of NaH (60% in mineral oil, 0.07 g, 1.82 mmol) in *N*, *N*-dimethylformamide (2 mL) was added compound **10** (0.32 g, 0.73 mmol) in *N*, *N*-dimethylformamide (3 mL) dropwise under argon atmosphere at room temperature and stirred for 30 min. Compound **23** (0.16 g, 0.61 mmol) in *N*, *N*-dimethylformamide (3 mL) was added dropwise to the above mixture. The resulting mixture was warmed to room temperature and stirred for 2h. After completion of the reaction, cooled to 0 ºC, quenched with H_2O (3 mL) and extracted with EtOAc (3 x 25 mL). The combined organic layer was washed with H_2O (3 x 15 mL) and brine (15 mL), dried over anhyd. Na_2SO_4 and concentrated *in vacuo*. The crude was purified by column chromatography (EtOAc/hexane = 1/4) to afford the desired product **33** (0.33 g, 81.8 %) as pale yellow solid. R_f = 0.6 (EtOAc/hexane = 1/2); ^1H NMR (300 MHz, $CDCl_3$) δ 9.05 (1H, s), 7.62 (1H, d, J = 15.3 Hz), 7.57–7.17 (20H, m), 6.81 (2H, s), 6.53 (1H, s) 5.06 (2H, s), 4.99 (4H, s); ^{13}C NMR (75 MHz, $CDCl_3$) δ 160.0, 157.3, 151.0, 147.7, 139.2, 136.9, 136.3, 131.9, 130.7, 128.9, 128.6, 128.4, 128.3, 128.1,

127.7, 127.33, 127.29, 127.1, 123.6, 118.4, 106.7, 106.3, 102.2, 70.2, 70.1; EI-MS *m/z* 549 ([M]$^+$, base), 458, 367.

(*E*)-6-(Benzyloxy)-2-(3,5-bis(benzyloxy)styryl)quinoline (34)

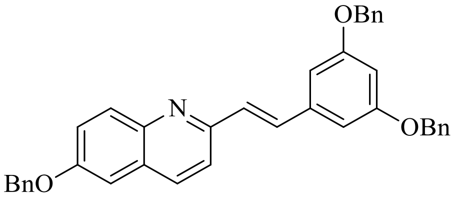

34

To a stirred solution of compound **18** (0.35 g, 1.40 mmol) and 3,5-dibenzyloxybenzaldehyde (0.54 g, 1.68 mmol) in anhyd. toluene (6 mL) were added $Fe(OAc)_2$ (0.01 g, 0.07 mmol) and trifluoroacetic acid (0.01 mL, 0.14 mmol) under argon atmosphere at room temperature. The reaction mixture was refluxed for 24 h. After completion of the reaction, cooled to room temperature and diluted with aqueous saturated $NaHCO_3$ solution. Two layers separated and the aqueous layer was extracted with EtOAc (3 x 25 mL). The combined organic layer was washed with H_2O (2 x 20 mL) and brine (20 mL), dried over anhyd. Na_2SO_4 and concentrated *in vacuo*. The crude was purified by column chromatography (EtOAc/hexane = 1/10) to afford the desired product **34** (0.43 g, 55.8 %) as orange solid. R_f = 0.4 (EtOAc/hexane = 1/4); ^{1}H NMR (300 MHz, $CDCl_3$) δ 7.95 (1H, d, *J* = 9.0 Hz), 7.87 (1H, d, *J* = 8.4 Hz), 7.50–7.25 (19H, m), 7.03 (1H, d, *J* = 2.7 Hz), 6.56 (2H, t, *J* = 2.1 Hz), 6.84 (1H, t, *J* = 2.1 Hz) 5.08 (2H, s), 5.01 (4H, s); ^{13}C NMR (75 MHz, $CDCl_3$) δ 160.0, 156.6, 153.4, 144.2, 138.7, 136.8, 136.4, 134.8, 133.0, 130.6, 129.5, 129.0, 128.44, 128.39, 128.1, 127.9, 127.8, 127.3, 122.4, 119.4, 106.7, 106.3, 102.8, 70.2, 70.1; EI-MS *m/z* 549 ([M]$^+$, base), 458, 367.

(*E*)-6-(Benzyloxy)-2-(3,5-bis(benzyloxy)styryl)quinoxaline (35)

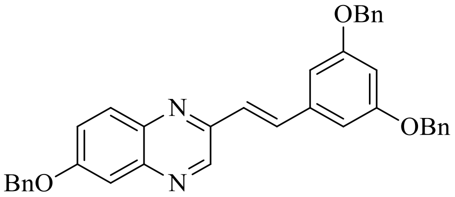

35

To a stirred solution of compound **29** (0.88 g, 3.52 mmol) and compound **9** (1.34 g, 4.22 mmol) in anhyd. toluene (10 mL) were added $Fe(OAc)_2$ (0.03 g, 0.18 mmol) and trifluoroacetic acid (0.03 mL, 0.35 mmol) under argon atmosphere at room temperature. The reaction mixture was refluxed for 24 h. After completion of the reaction, cooled to room temperature and diluted with aqueous saturated $NaHCO_3$ solution. Two layers were separated and the aqueous layer was extracted with EtOAc (3 x 25 mL). The combined organic layer was washed with H_2O (2 x 20 mL) and brine (20 mL), dried over anhyd. Na_2SO_4 and concentrated *in vacuo*. The crude was purified by column chromatography (EtOAc/hexane = 1/8) to afford the desired product **35** (1.25 g, 64.4 %) as pale yellow solid. R_f = 0.4 (EtOAc/hexane = 1/1); ^{1}H NMR (300 MHz, $CDCl_3$) δ 8.89 (1H, s), 7.92 (1H, d, *J* = 9.0 Hz), 7.66 (1H, d, *J* = 16.2 Hz), 7.48–7.29 (17H, m), 7.26 (1H, d, *J* = 16.2 Hz), 6.86 (2H, d, *J* = 2.1 Hz), 6.60 (1H, t, *J* = 2.1 Hz), 5.19 (2H, s), 5.06 (4H, s); ^{13}C NMR (75 MHz, $CDCl_3$) δ 160.3, 159.4, 148.3, 144.3, 143.1, 138.6, 138.3, 136.8, 136.2, 134.8, 130.2, 128.6, 128.5, 128.2, 127.9, 127.5, 127.4, 126.0, 123.6, 108.3, 106.7, 103.4, 70.7, 70.4; EI-MS *m/z* 550 ([M]$^+$, base), 459, 368.

(*E*)-6-(Benzyloxy)-2-(3,5-bis(benzyloxy)styryl)quinazoline (36)

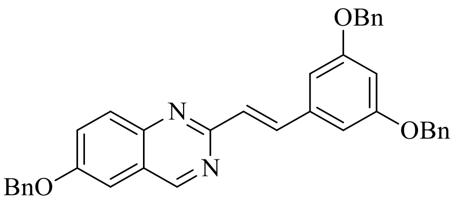

36

To a stirred solution of compound **30** (0.38 g, 1.522 mmol) and compound **9** (0.58 g, 1.823 mmol) in anhyd. DMF (12 mL) was added ^{t}BuOK (0.26 g, 2.283 mmol) under nitrogen atmosphere. The resulting mixture was stirred for 17 h at room temperature. After completion of the reaction, H_2O (4 mL) was added dropwise to quench the excess ^{t}BuOK. The reaction mixture was extracted with EtOAc (3 x 30 mL). The combined organic layer was washed with H_2O (4 x 20 mL) and brine (2 x 20 mL), dried over anhyd. Na_2SO_4 and the solvent was removed under reduced pressure. The crude was purified by column chromatography (EtOAc/hexane = 1/5–1/3) to afford the desired product **36** (0.5 g, 59.8%) as pale yellow color solid. R_f = 0.56 (EtOAc/hexane = 1/3); ^{1}H NMR (300 MHz, $CDCl_3$): δ 9.15 (1H, s), 7.94 (1H, d, *J* = 15.9 Hz) 7.86 (1H, d, *J* = 9.0 Hz), 7.55–7.26 (17H, m), 7.10 (1H, s), 6.88 (2H, s),

6.58 (1H, d, J = 1.5 Hz), 5.11 (2H, s), 5.02 (4H, s); ^{13}C NMR (75 MHz, $CDCl_3$): δ 160.0, 159.3, 158.3, 157.0, 146.5, 138.3, 136.9, 136.7, 135.9, 129.5, 128.5, 128.3, 128.0, 127.7, 127.2, 127.0, 124.0, 106.6, 105.7, 103.3, 70.4, 70.1; EI-MS m/z 550 ($[M]^+$, base), 460, 368.

(*E*)-5-(2-(6-hydroxynaphthalen-2-yl)vinyl)benzene-1,3-diol (2)

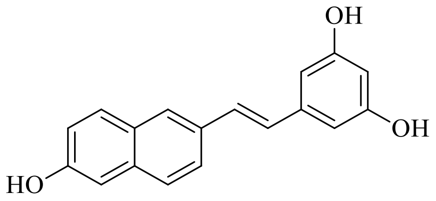

2

^{1}H NMR (300 MHz, CD_3OD) δ 7.71–7.56 (4H, m), 7.14 (1H, d, J = 16.2 Hz), 7.02 (1H, d, J = 16.2 Hz), 7.07–7.02 (2H, m), 6.53 (2H, d, J = 2.1 Hz), 6.21 (1H, t, J = 2.2 Hz); ^{13}C NMR (75 MHz, CD_3OD) δ 159.5, 156.4, 141.0, 135.9, 133.4, 130.4, 129.9, 129.8, 128.8, 127.5, 127.3, 124.8, 119.4, 110.1, 106.1, 103.1; HRMS m/z (M^+) calcd for $C_{18}H_{14}O_3$: 278.0943, Found: 278.0941.

Synthesis of (*E*)-5-(2-(7-hydroxyquinolin-3-yl)vinyl)benzene-1,3-diol (3)

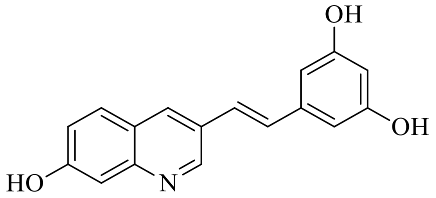

3

To a stirred solution of compound **32** (1.07 g, 1.95 mmol) in anhyd. CH_2Cl_2 (10 mL) was added BBr_3 (1.0 M in CH_2Cl_2, 7.79 mL) dropwise at -20 ºC under argon atmosphere and stirred for 2 h. After completion of the reaction, MeOH (3.5 mL) was added dropwise to quench the excess BBr_3, warmed to room temperature in 20 min and the solvent was removed under reduced pressure. H_2O (15 mL) was added to the crude residue and extracted with EtOAc (3 x 50 mL). The combined organic layer was washed with H_2O (3 x 20 mL) and brine (20 mL), dried over anhyd. Na_2SO_4 and concentrated *in vacuo*. The crude was purified by column chromatography ($MeOH/CH_2Cl_2$ = 1/19) to afford the desired product **3** (0.47 g, 87.0 %) as pale orange solid. mp 262 ºC; R_f = 0.37 ($MeOH/CH_2Cl_2$ = 1/9); ^{1}H NMR (300 MHz, CD_3OD) δ 9.08 (1H, s), 9.01 (1H, s) 8.11 (1H, d, J = 9.3 Hz), 7.40 (1H, dd, J = 9.3, 2.1

Hz), 7.32 (1H, d, *J* = 2.1 Hz), 7.26 (1H, d, *J* = 16.5 Hz), 7.12 (1H, d, *J* = 16.5 Hz), 6.44 (2H, d, *J* = 1.8 Hz), 6.07 (1H, d, *J* = 1.8 Hz); ^{13}C NMR (75 MHz, CD_3OD) δ 159.2, 158.6, 148.9, 148.3, 139.2, 132.9, 130.2, 129.4, 128.4, 124.7, 123.1, 119.9, 109.0, 105.2, 102.5; HRMS *m/z* (M^+) calcd for $C_{17}H_{13}NO_3$: 279.0895, Found: 279.0892; UV-Vis (MeOH): λ_{abs} 240, 287, 337, 391.

(*E*)-5-(2-(6-Hydroxyquinolin-2-yl)vinyl)benzene-1,3-diol (4)

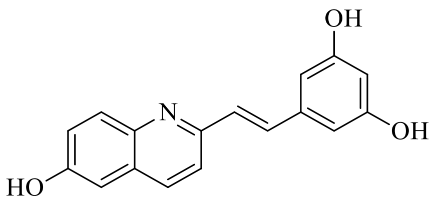

4

To a stirred solution of compound **34** (0.25 g, 0.45 mmol) in anhyd. CH_2Cl_2 (10 mL) was added BBr_3 (1.0 M in CH_2Cl_2, 1.82 mL) dropwise at -20 ºC under argon atmosphere. The reaction mixture was warmed to room temperature and stirred for 2 h. After completion of the reaction, cool to 0 ºC, MeOH (2 mL) was added dropwise to quench the excess BBr_3, warmed to room temperature in 20 min and the solvent was removed under reduced pressure. H_2O (10 mL) was added to the crude residue and extracted with EtOAc (3 x 40 mL). The combined organic layer was washed with H_2O (3 x 20 mL) and brine (20 mL), dried over anhyd. Na_2SO_4 and concentrated *in vacuo*. The crude was purified by column chromatography ($MeOH/CH_2Cl_2$ = 1/19) to afford the desired product **4** (0.08 g, 61.3 %) as pale orange solid. mp 182 ºC (decomposed); R_f = 0.3 ($MeOH/CH_2Cl_2$ = 1/9); ^1H NMR (300 MHz, CD_3OD) δ 8.06 (1H, d, *J* = 8.7 Hz), 7.83 (1H, d, *J* = 9.3 Hz), 7.76 (1H, d, *J* = 8.7 Hz), 7.44 (1H, d, *J* = 16.2 Hz), 7.30 (1H, dd, *J* = 9.3, 2.7 Hz), 7.22 (1H, d, *J* = 16.2 Hz), 7.10 (1H, d, *J* = 2.7 Hz), 6.58 (2H, d, *J* = 1.8 Hz), 6.26 (1H, t, *J* = 1.8 Hz); ^{13}C NMR (75 MHz, CD_3OD) δ 159.6, 156.9, 154.4, 143.6, 139.7, 136.6, 135.5, 130.0, 129.9, 128.7, 123.3, 120.0, 109.8, 106.8, 104.4; HRMS *m/z* (M^+) calcd for $C_{17}H_{13}NO_3$: 279.0895, Found: 279.0896; UV-Vis (MeOH): λ_{abs} 231, 308, 390.

(*E*)-5-(2-(7-hydroxyisoquinolin-3-yl)vinyl)benzene-1,3-diol (5)

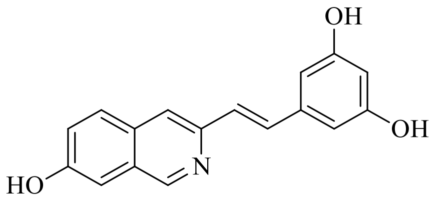

5

To a stirred solution of compound **33** (0.13 g, 0.24 mmol) in anhyd. CH_2Cl_2 (5 mL) was added BBr_3 (1.0 M in CH_2Cl_2, 0.95 mL) dropwise at -20 ºC under argon atmosphere. The reaction mixture was warmed to room temperature and stirred for 2 h. After completion of the reaction, cool to 0 ºC, MeOH (2 mL) was added dropwise to quench the excess BBr_3, warmed to room temperature in 20 min and the solvent was removed under reduced pressure. H_2O (10 mL) was added to the crude residue and extracted with EtOAc (3 x 35 mL). The combined organic layer was washed with H_2O (3 x 15 mL) and brine (15 mL), dried over anhyd. Na_2SO_4 and concentrated *in vacuo*. The crude was purified by column chromatography ($MeOH/CH_2Cl_2$ = 1/19) to afford the desired product **5** (0.04 g, 60.6 %) as pale yellow solid. R_f = 0.4 ($MeOH/CH_2Cl_2$ = 1/9); ^1H NMR (300 MHz, CD_3OD) δ 8.97 (1H, s), 7.78 (1H, d, J = 8.7 Hz), 7.74 (1H, s), 7.43 (1H, d, J = 15.6 Hz), 7.33 (1H, dd, J = 8.7, 2.4 Hz), 7.26 (1H, d, J = 2.4 Hz), 7.19 (1H, d, J = 15.6 Hz), 6.57 (2H, d, J = 2.4 Hz), 6.23 (1H, t, J = 2.4 Hz); ^{13}C NMR (75 MHz, CD_3OD) δ 159.8, 158.1, 151.5, 147.9, 140.4, 132.8, 132.0, 130.8, 129.6, 128.5, 125.0, 119.9, 109.5, 106.3, 103.6; HRMS *m/z* (M^+) calcd for $C_{17}H_{13}NO_3$: 279.0895, Found: 279.0894; UV-Vis (MeOH): λ_{abs} 240, 345.

(*E*)-5-(2-(6-hydroxyquinoxalin-2-yl)vinyl)benzene-1,3-diol (6)

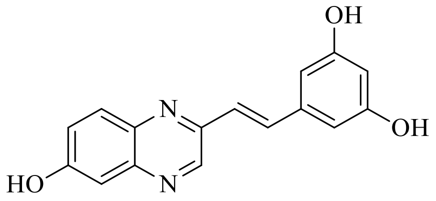

6

To a stirred solution of compound **35** (0.12 g, 0.22 mmol) in anhyd. CH_2Cl_2 (4 mL) was added BBr_3 (1.0 M in CH_2Cl_2, 0.87 mL) dropwise at -20 ºC under argon atmosphere. The reaction mixture was warmed to room temperature and stirred for 2 h. After completion of the reaction, cool to 0 ºC, MeOH (2 mL) was added dropwise to quench the excess BBr_3, warmed

to room temperature in 20 min and the solvent was removed under reduced pressure. H_2O (10 mL) was added to the crude residue and extracted with EtOAc (3 x 35 mL). The combined organic layer was washed with H_2O (3 x 15 mL) and brine (15 mL), dried over anhyd. Na_2SO_4 and concentrated *in vacuo*. The crude was purified by column chromatography ($MeOH/CH_2Cl_2$ = 1/19) to afford the desired product **6** (0.04 g, 75.5 %) as pale yellow solid. mp 318 ºC; R_f = 0.4 ($MeOH/CH_2Cl_2$ = 1/9); ^1H NMR (300 MHz, CD_3OD) δ 8.95 (1H, s), 7.80 (1H, d, *J* = 9.0 Hz), 7.59 (1H, d, *J* = 15.6 Hz), 7.36 (1H, dd, *J* = 9.0, 2.7 Hz), 7.23 (1H, d, *J* = 2.7 Hz), 7.21 (1H, d, *J* = 15.6 Hz), 6.61 (2H, d, *J* = 1.8 Hz), 6.28 (1H, t, *J* = 1.8 Hz); ^{13}C NMR (75 MHz, CD_3OD) δ 159.7, 159.3, 149.1, 145.1, 143.3, 139.1, 138.3, 136.2, 130.5, 125.6, 124.0, 110.2, 106.8, 104.5; HRMS *m/z* (M^+) calcd for $C_{16}H_{12}N_2O_3$: 280.0848, Found: 280.0847; UV-Vis (MeOH): λ_{abs} 219, 323, 430.

(*E*)-5-(2-(6-hydroxyquinazolin-2-yl)vinyl)benzene-1,3-diol (7)

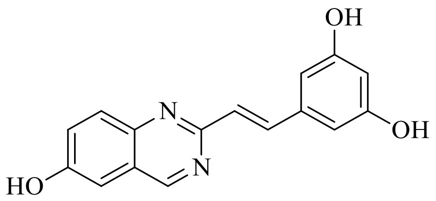

7

To a stirred solution of compound **36** (0.05 g, 0.1 mmol) in anhyd. CH_2Cl_2 (4 mL) was added BBr_3 (0.1 mL, 1.0 mmol) dropwise at -20 ºC under nitrogen atmosphere and the resulting mixture was stirred for 22 h. After completion of the reaction, solvent was removed *in vacuo*. To the crude, H_2O (3.5 mL) was added and stirred for 10–15 min. The reaction mixture was extracted with THF/EtOAc (1/20) (3 x 25 mL). The combined organic layer was washed with H_2O (3 x 15 mL) and brine (2 x 15 mL), dried over anhyd. Na_2SO_4 and concentrated *in vacuo*. The crude was purified by column chromatography ($MeOH/CH_2Cl_2$ = 1/20–1/10) to afford the desired product **7** (0.01 g, 35.7%) as pale yellow color solid. R_f = 0.32 ($MeOH/CH_2Cl_2$ = 1/10). ^1H NMR (400MHz, DMSO-d_6) δ 10.46 (1H, s), 9.43 (2H, s), 9.37 (1H, s), 7.84 (1H, d, *J* = 7.2 Hz), 7.78 (1H, d, *J* = 15.6 Hz), 7.53 (1H, dd, *J* = 7.2, 2.8 Hz), 7.28 (1H, d, *J* = 2.8 Hz), 7.12 (1H, d, *J* = 15.6 Hz), 6.55 (2H, d, *J* = 2.0 Hz), s), 6.25 (1H, t, *J* = 2.0 Hz); ^{13}C NMR (100 MHz, DMSO-d_6) δ 158.9, 158.8, 158.0, 156.3, 144.8, 137.6, 136.6, 129.2, 127.6, 127.0, 124.4, 108.0, 105.5, 103.6; EI-MS *m/z* 281 (M^+ + H, base), 280 (M^+), 263, 251; HRMS *m/z* (M^+) calcd for $C_{16}H_{12}N_2O_3$: 280.0848, Found: 280.0844; UV-Vis (MeOH): λ_{abs} 236, 348.

Antioxidant assay using ABTS

The radical cation was prepared by mixing an equal amount of 7mM ABTS (2,2'-azino-bis(3-ethylbenzothiazoline-6-sulphonic acid)) stock solution with 2.45 mM potassium persulfate stock solution. This mixture was stored at 0˚C for 12 hours under darkness. The above ABTS solution was diluted with methanol appropriately to give UV absorption value of 1.000 at the 734 nm. The compounds **1-7** were dissolved in methanol to prepare 1000 µM stock solutions. Then these solutions were diluted with methanol to 500 µM, 250 µM, 125 µM, 62.5 µM, 31.25 µM, 15.63 µM, 7.81 µM, 3.90 µM, 1.95 µM, and 0.97 µM. In different 3 sets of test tubes, 0.9 mL of the ABTS solution and 0.1 mL of the compound solution were mixed under darkness. After 30 min of incubation, the UV absorption at 734 nm was measured. Control was used the mixture of 0.9 mL ABTS and 0.1 mL methanol. The radical scavenging rates were obtained from these UV absorption data and the resulting IC_{50} values were calculated using Origin 8.

NO assay and cell viability test

Murine macrophage, Raw 264.7 cells were grown in Dulbecco's modified Eagle's medium (Gibco, Carlsbad , CA, USA), supplemented with 2 mM glutamine, antibiotics (100 U/mL of penicillin A and 100 mg/mL of streptomycin) and 10% FBS (Gibco). Cells were cultured in 96-well plates (5×10^4 cells), treated with serial dilutions of compound (3.125-100 µM) and stimulated with LPS (1 µg/mL) for 24 h at 37°C. After incubation, the supernatant was collected and the amount of nitrite was quantified using the Griess Reagent System (Promega, Fitchburg, WI, USA). For the correction of NO production, cell viability was measured using the CellTiter 96® Aqueous one solution cell proliferation assay kit (Promega). Cells were incubated with varying amounts of compound for 24 h, then MTS (tetrazolium salt) reagent was added to the plates. After 1h, the absorbance at 490 nm was read using a microplate reader (Thermo Scientific, Waltham, MA, USA).

Statistical analysis. Statistical significance was analyzed using the GraphPad Prism 5 software (GraphPad Software, San Diego, CA, USA). The comparison of two groups was performed by the unpaired *t* test. Values of **P* less than 0.05, ***P* less than 0.01 and ****P* less than 0.001 were considered statistically significant.

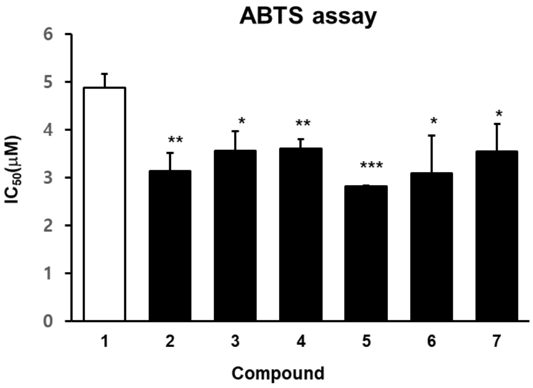

Fig. S1: ABTS assay of compounds **1**–**7** (Statistical analysis).

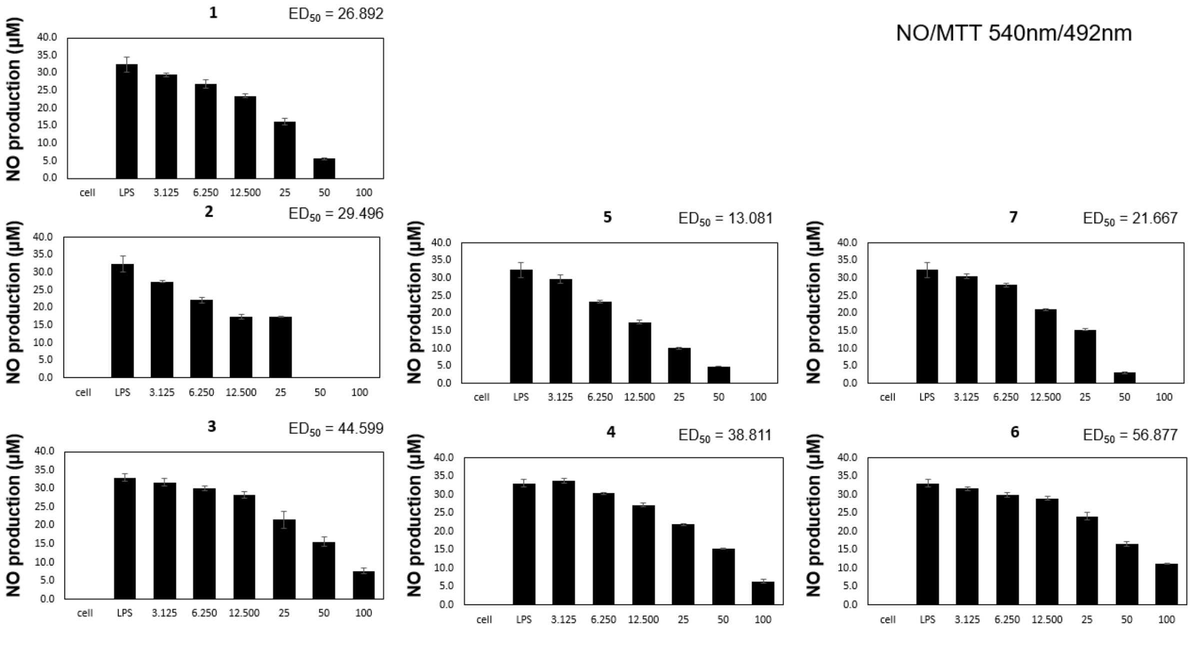

Fig. S2: Inhibitory effect of compounds **1**–**7** on NO production.

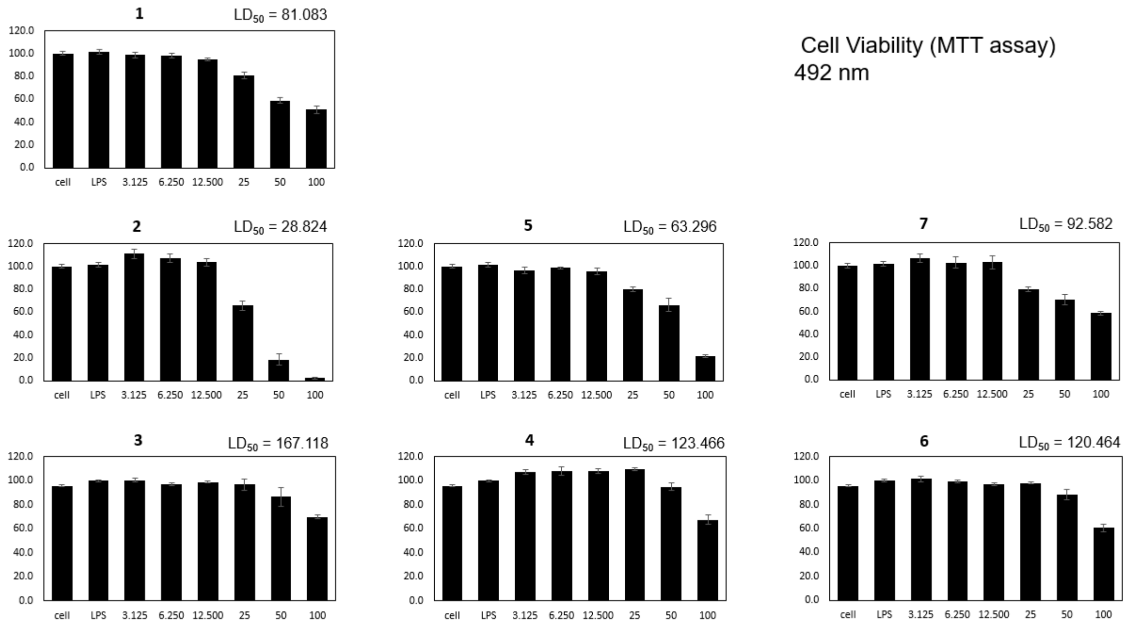

Fig. S3: Cell viability (MTT) assay of compounds **1**–**7**.

Reverse transcriptase-polymerase chain reaction (RT-PCR)

Total RNA was isolated from Raw 264.7 cells using TRIzol reagent (Invitrogen, Waltham, MA, USA). Relative levels of pro-inflammatory cytokine gene expression were tested by 1.5% agarose gel electrophoresis after endpoint RT-PCR using GoScript™ Reverse Transcriptase (Promega). The sequences of the sense and anti-sense primers used for amplification are as follows. IL-1β, 5'-CCTGTGTAATGAAAGACGGCACAC-3', 5'-CTTGTGAGGTGCTGATGTACCAGT-3'; IL-6, 5'-GCCAGAGTCCTTCAGAGAGATACA-3', 5'-ATTGGATGGTCTTGGTCCTTAGCC-3'; TNF-α, 5'-TCTCATCAGTTCTATGGCCCAGAC-3', 5'-GGCACCA CTAGTTGGTTGTCTTTG-3'. As a control, glyceraldehyde-3-phosphate dehydrogenase (GAPDH) gene was also amplified using 5'-GACATCAAGAAGGTGGTGAAGCAG-3', 5'-CCCTGTTGCTGTAGCCGTATTCAT-3'.

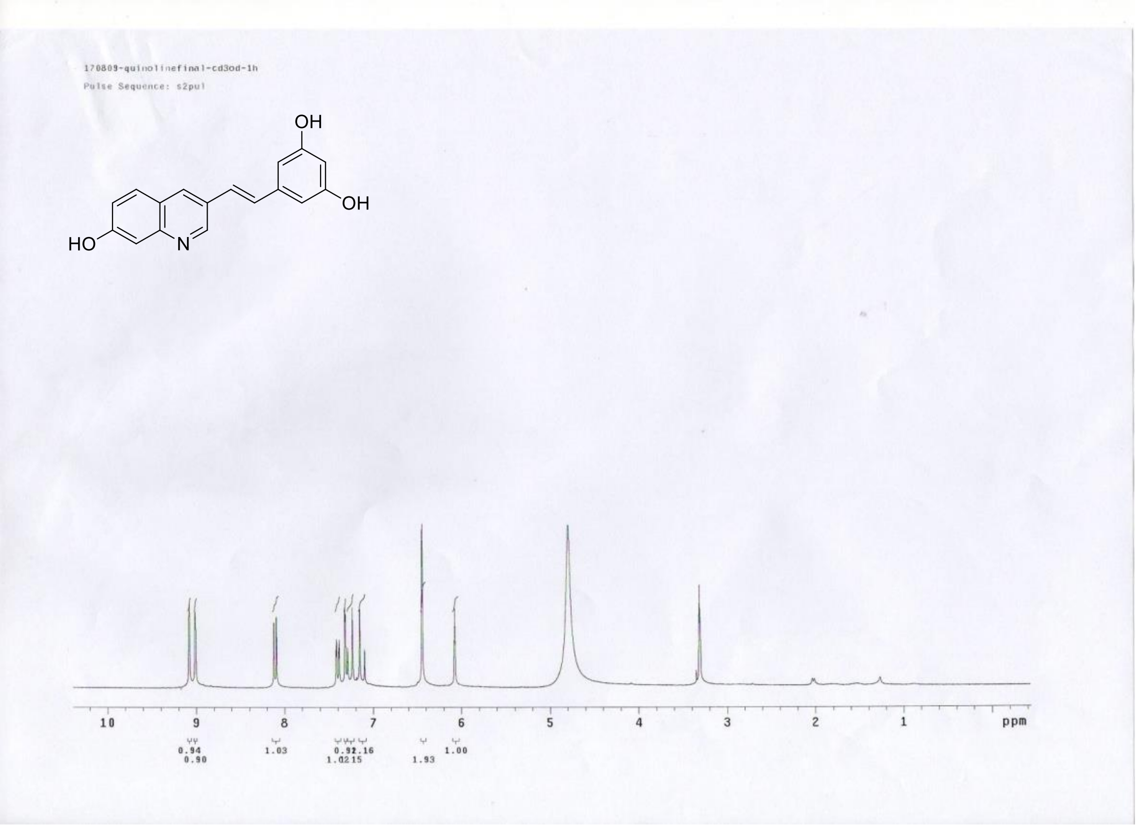
170809-quinolinefinal-cd3od-1h
Pulse Sequence: s2pul
OH
OH
HO
N
10
9
8
7
6
5
4
3
2
1
ppm
0.94
0.90
1.03
0.92.16
1.02.15
1.93
1.00

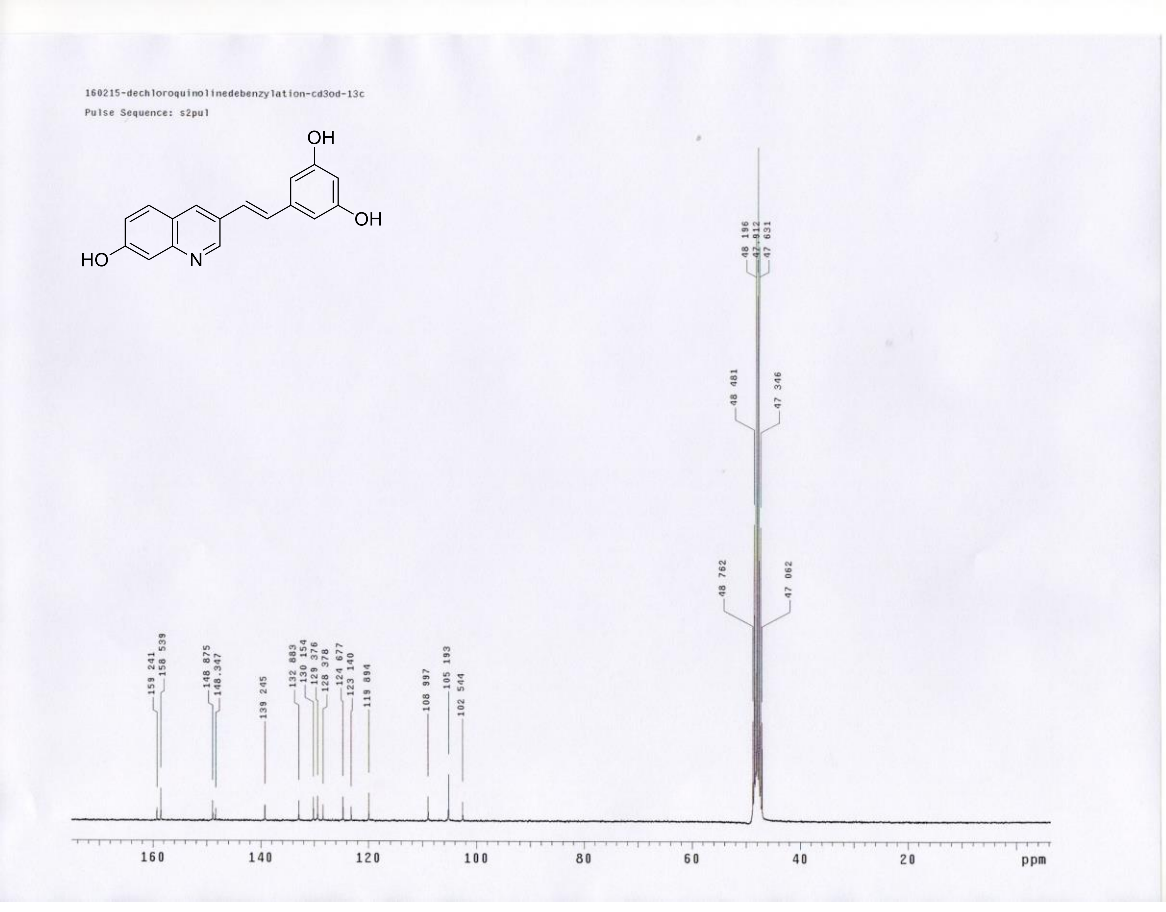
160215-dechloroquinolinedebenzylation-cd3od-13c
Pulse Sequence: s2pul
OH
OH
HO
N
48 196
47 912
47 631
48 481
47 346
48 762
47 062
159 241
158 539
148 875
148.347
139 245
132 883
130 154
129 376
128 378
124 677
123 140
119 894
108 997
105 193
102 544
160
140
120
100
80
60
40
20
ppm

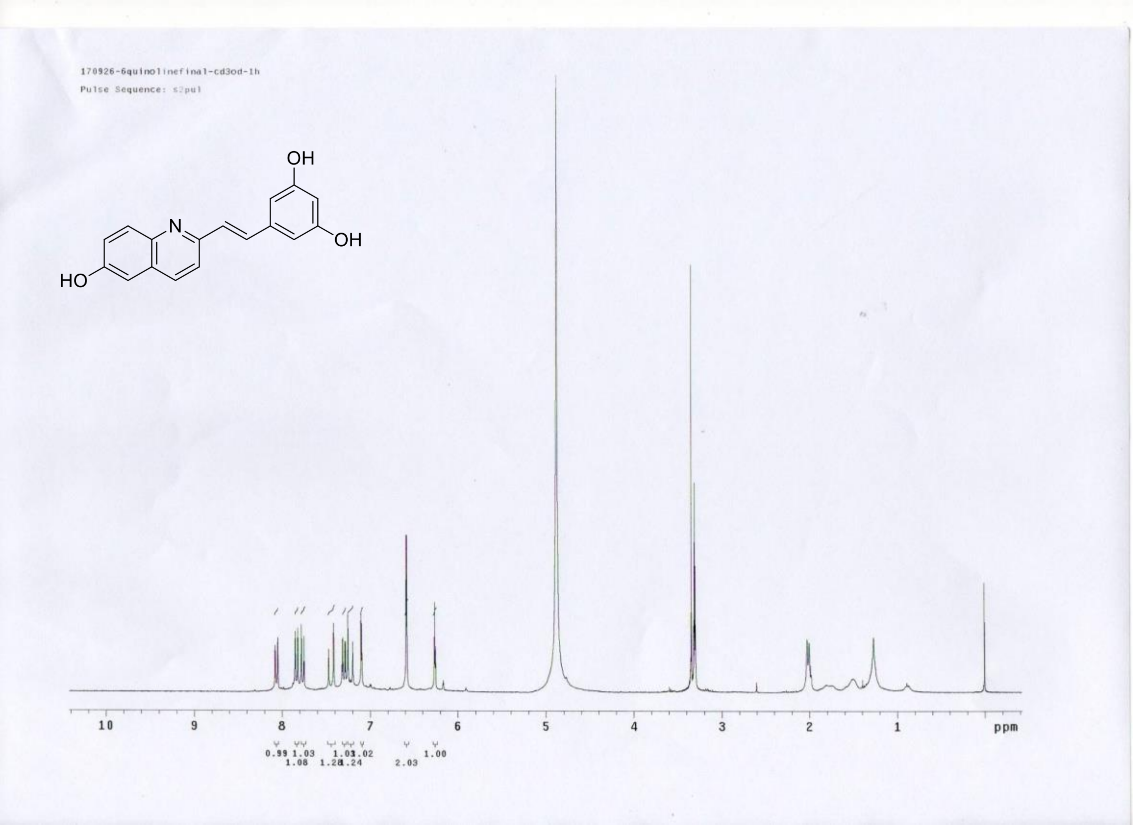
170926-6quinolinefinal-cd3od-1h
Pulse Sequence: s2pul
OH
N
OH
HO
ppm

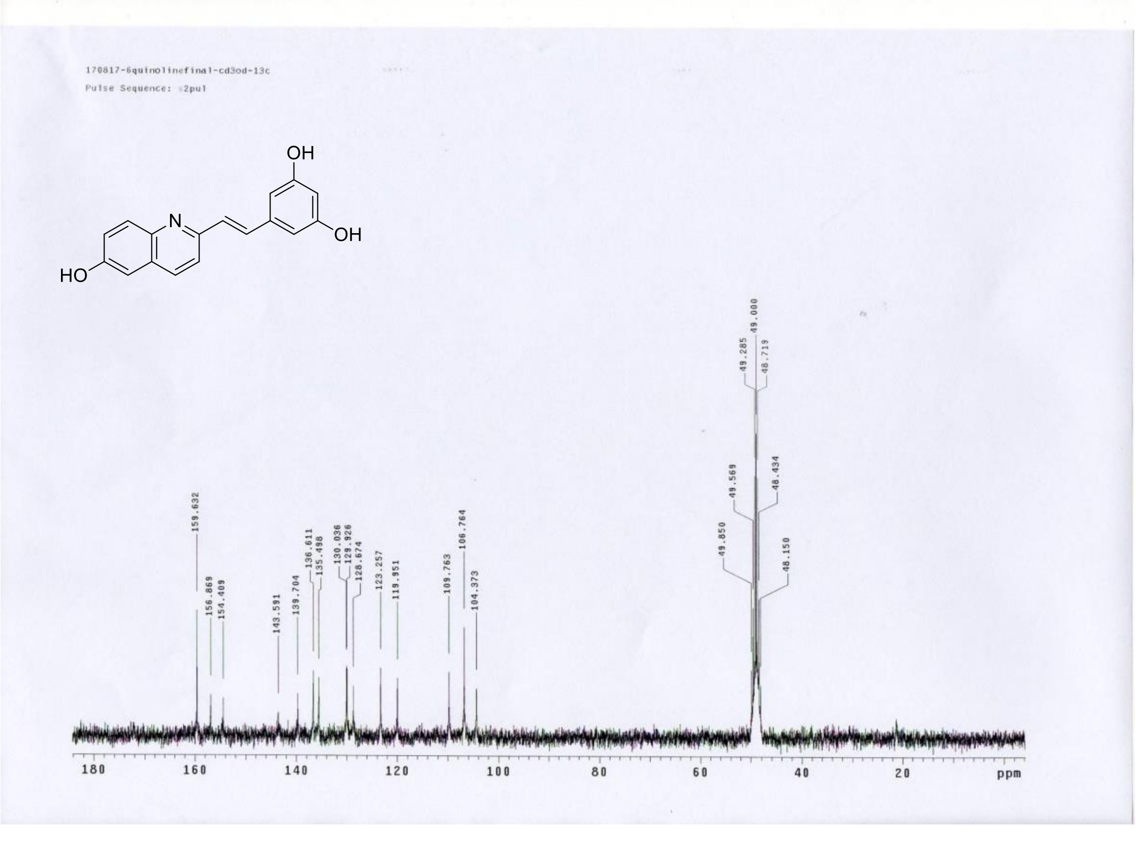
170817-6quinolinefinal-cd3od-13c
Pulse Sequence: s2pul
OH
N
OH
HO
159.632
156.869
154.409
143.591
139.704
136.611
135.498
130.036
129.926
128.674
123.257
119.951
109.763
106.764
104.373
49.850
49.569
49.285
49.000
48.719
48.434
48.150
ppm

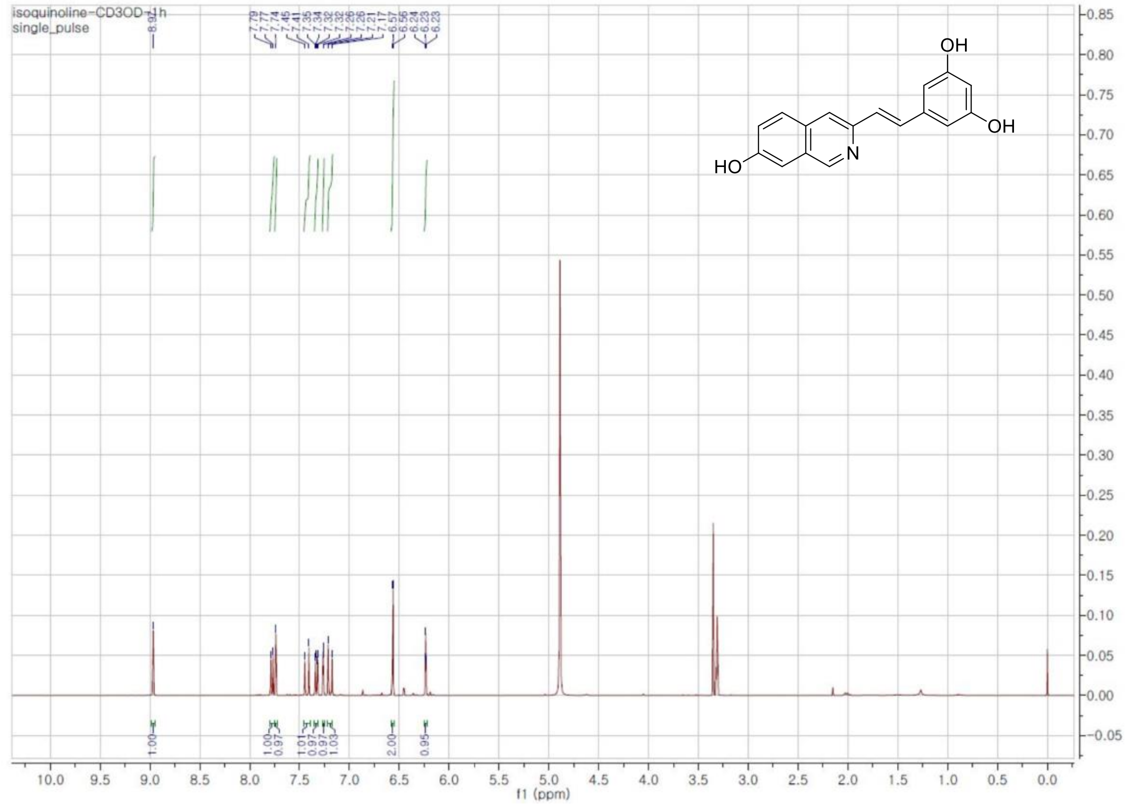

isoquinoline-CD3OD-1h
single_pulse
OH
OH
HO
N
f1 (ppm)

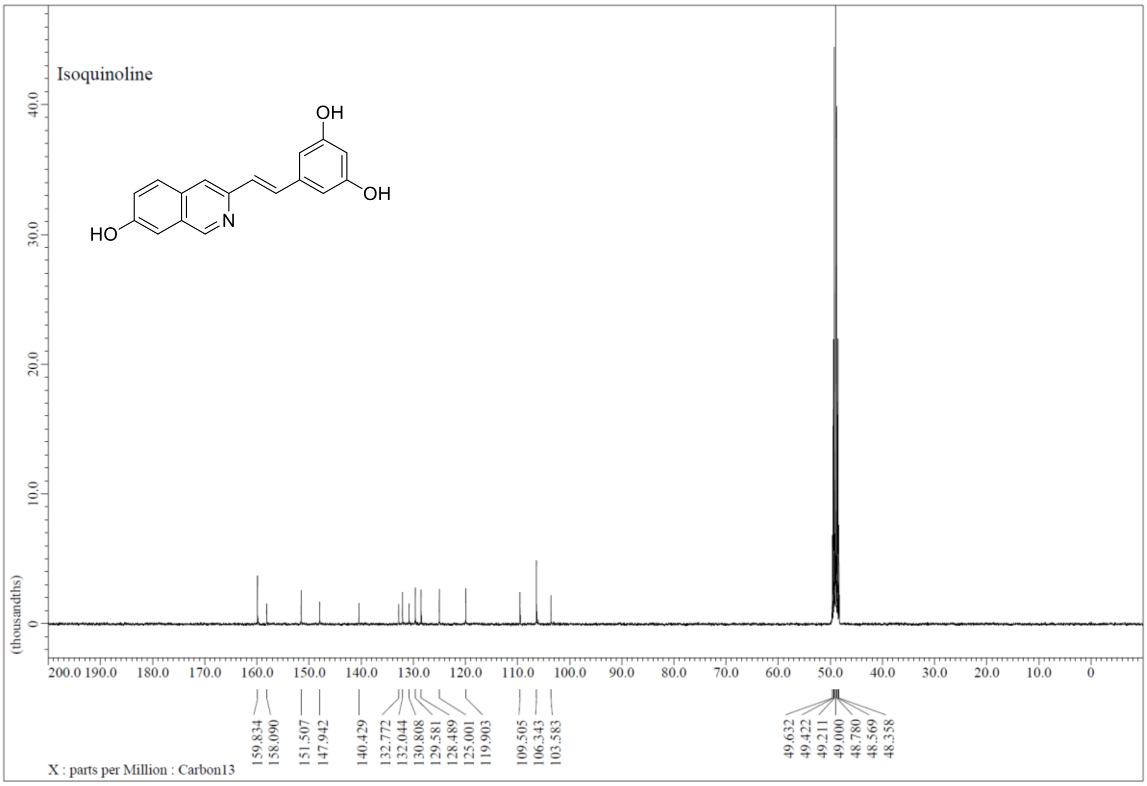

Isoquinoline
OH
OH
HO
N
(thousandths)
159.834
158.090
151.507
147.942
140.429
132.772
132.044
130.808
129.581
128.489
125.001
119.903
109.505
106.343
103.583
49.632
49.422
49.211
49.000
48.780
48.569
48.358
X : parts per Million : Carbon13

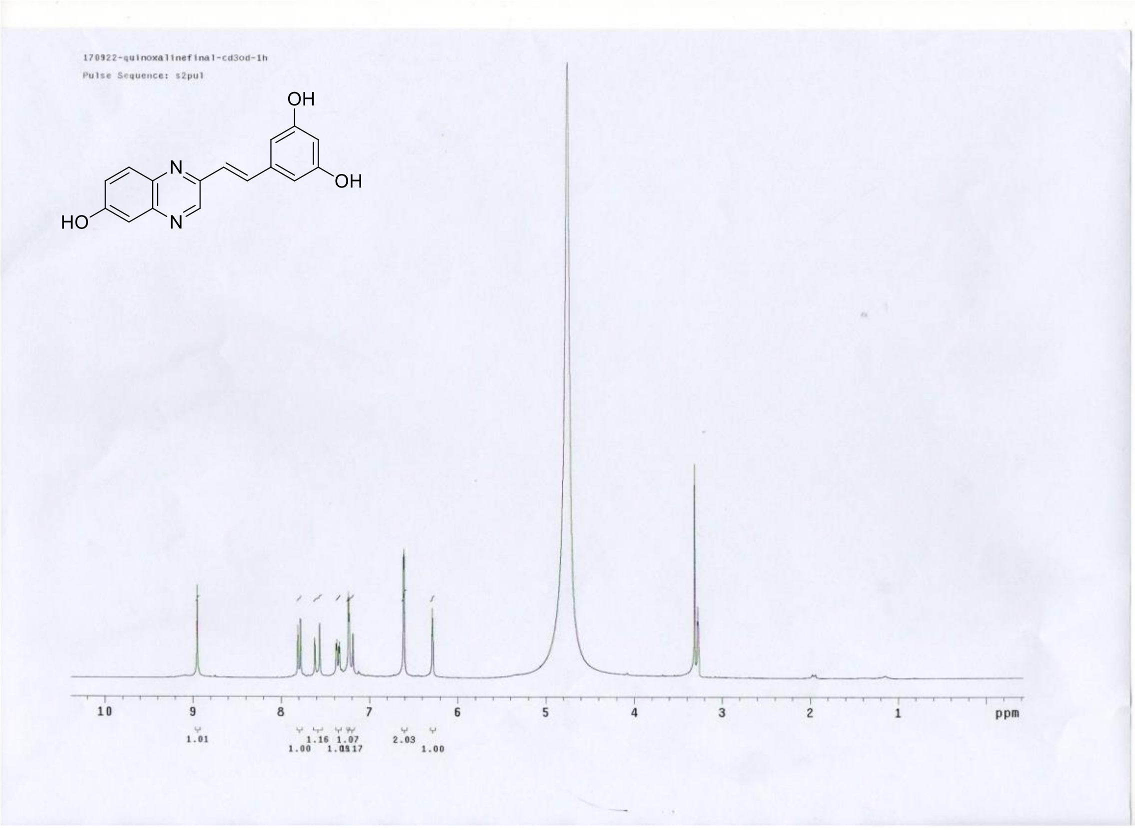
170922-quinoxalinefinal-cd3od-1h
Pulse Sequence: s2pul
OH
N
N
OH
HO
ppm
1.01
1.16
1.07
1.00
2.03
1.00

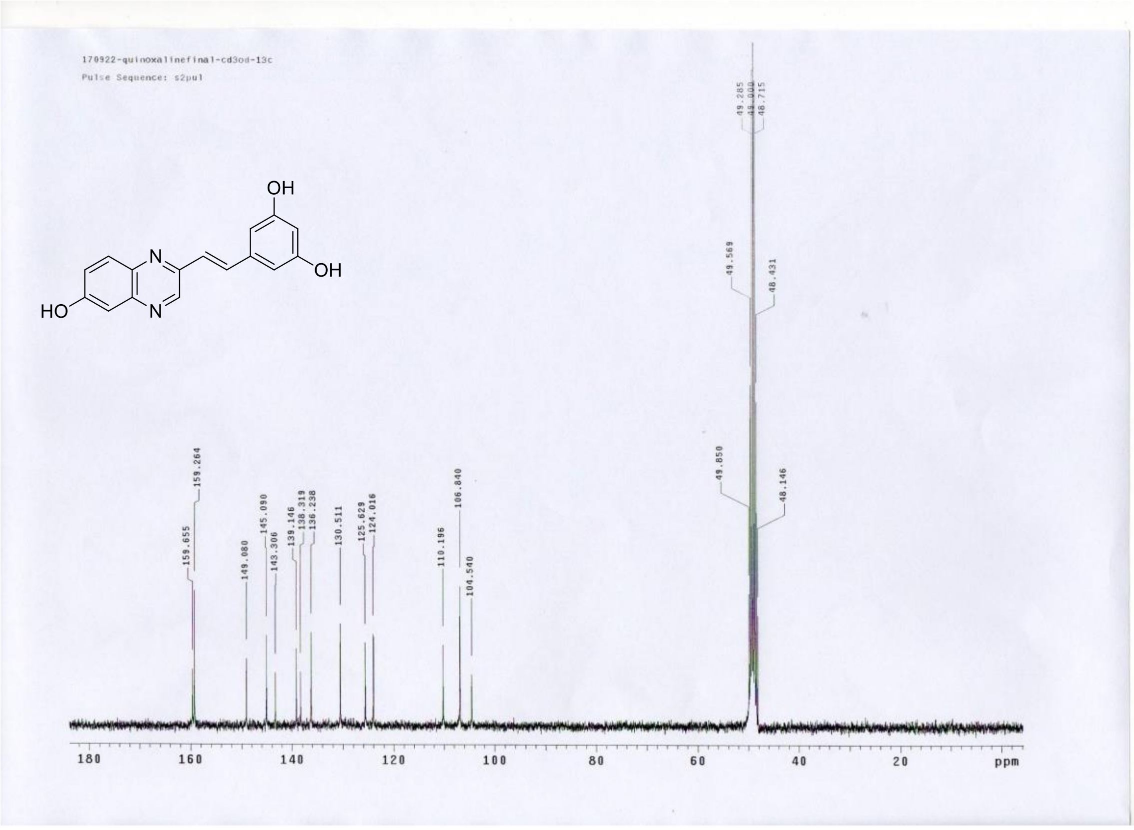
170922-quinoxalinefinal-cd3od-13c
Pulse Sequence: s2pul
OH
N
N
OH
HO
159.655
159.264
149.080
145.090
143.306
139.146
138.319
136.298
130.511
125.629
124.016
110.196
106.840
104.540
49.850
49.569
49.285
48.715
48.431
48.146
ppm

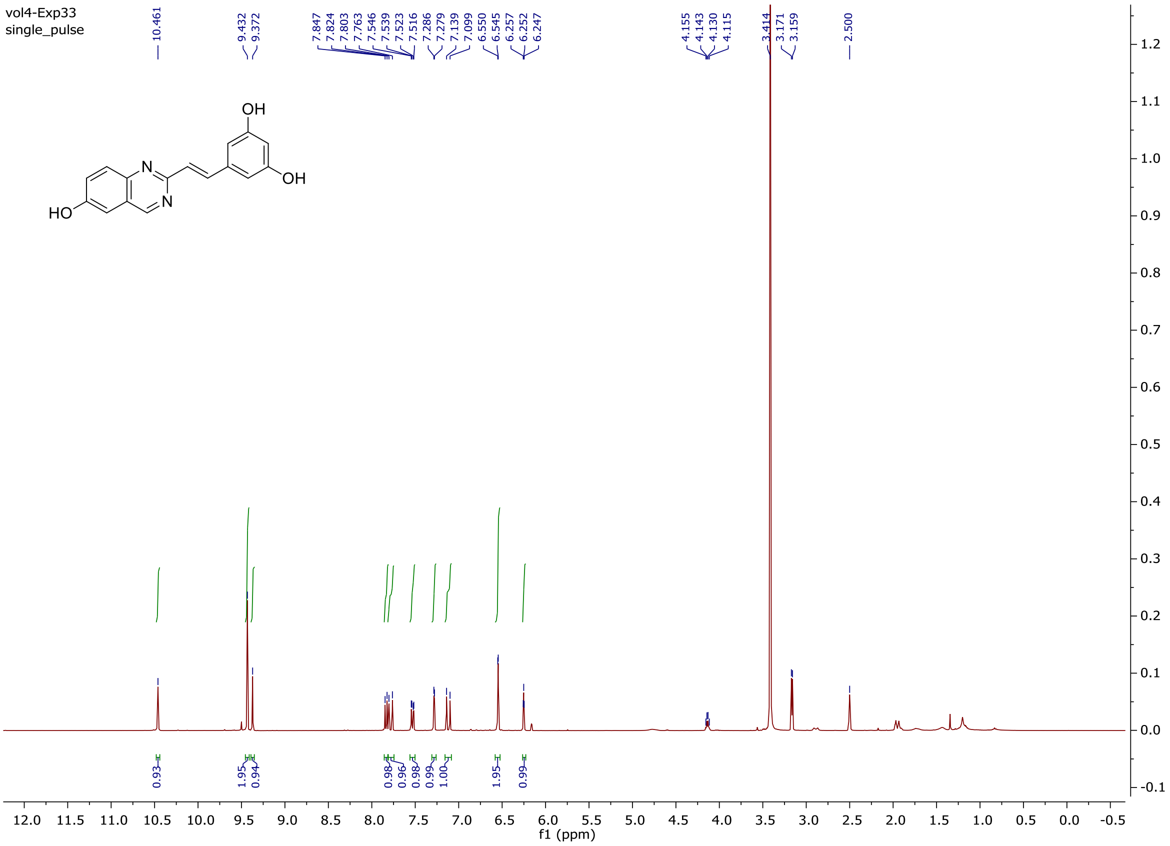
vol4-Exp33
single_pulse
10.461
9.432
9.372
7.847
7.824
7.803
7.763
7.546
7.539
7.523
7.516
7.286
7.279
7.139
7.099
6.550
6.545
6.257
6.252
6.247
4.155
4.143
4.130
4.115
3.414
3.171
3.159
2.500
OH
N
N
OH
HO
0.93
1.95
0.94
0.98
0.96
0.98
0.99
1.00
1.95
0.99
f1 (ppm)

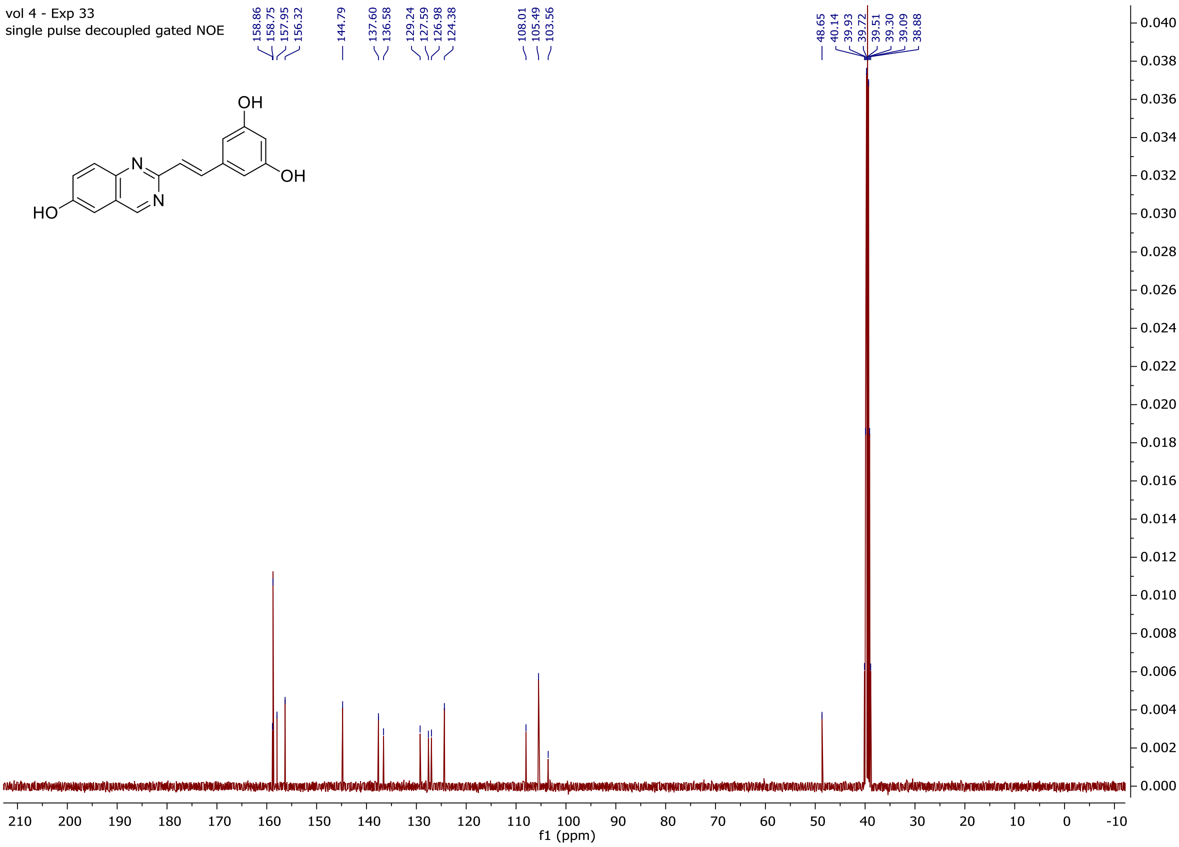
vol 4 - Exp 33
single pulse decoupled gated NOE
158.86
158.75
157.95
156.32
144.79
137.60
136.58
129.24
127.59
126.98
124.38
108.01
105.49
103.56
48.65
40.14
39.93
39.72
39.51
39.30
39.09
38.88
OH
N
N
OH
HO
f1 (ppm)

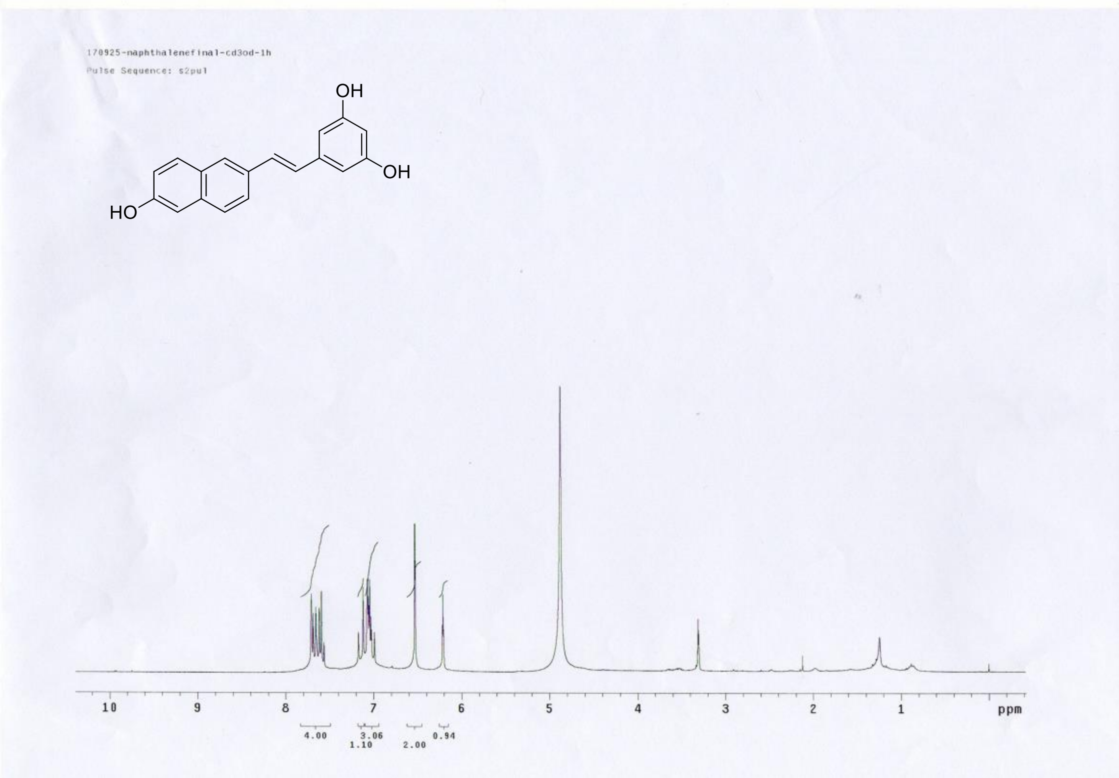
170925-naphthalenefinal-cd3od-1h
Pulse Sequence: s2pul
OH
OH
HO
10 9 8 7 6 5 4 3 2 1 ppm
4.00
3.06
1.10
2.00
0.94

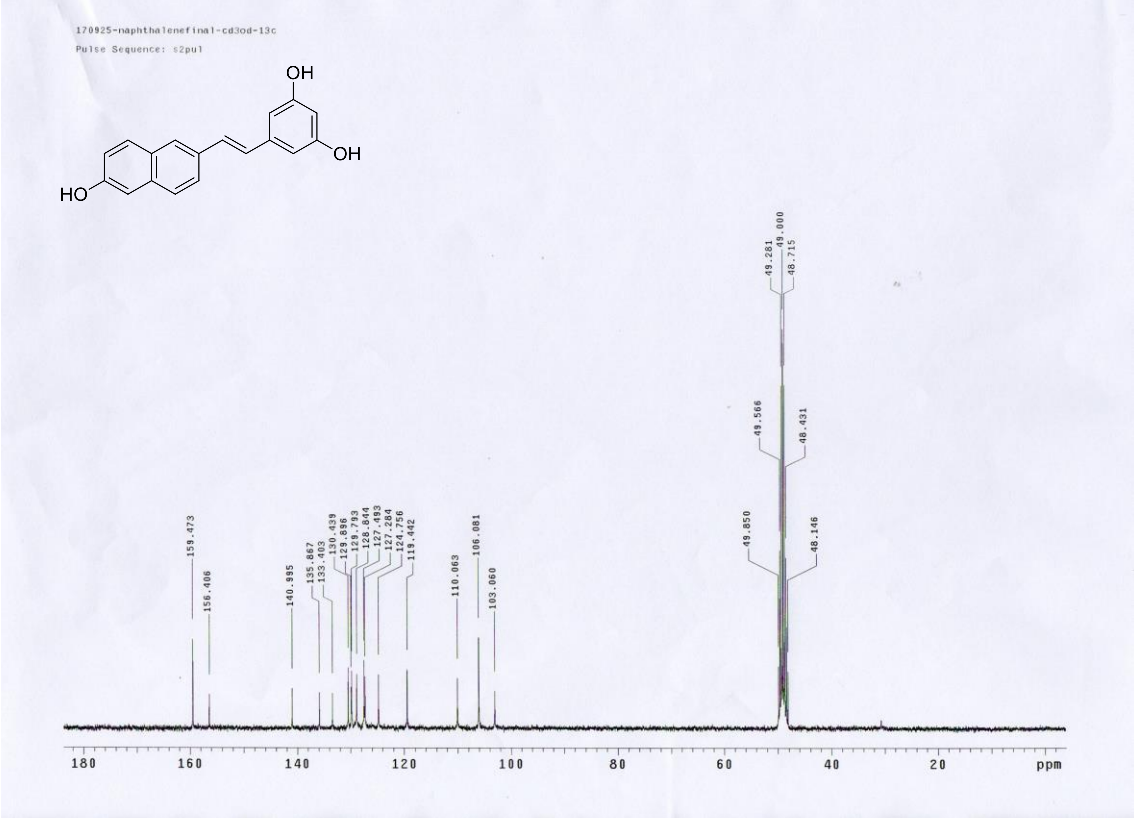
170925-naphthalenefinal-cd3od-13c
Pulse Sequence: s2pul
OH
OH
HO
159.473
156.406
140.995
135.867
133.403
130.439
129.896
129.793
128.844
127.493
127.284
124.756
119.442
110.063
106.081
103.060
49.850
49.566
49.281
49.000
48.715
48.431
48.146
180 160 140 120 100 80 60 40 20 ppm

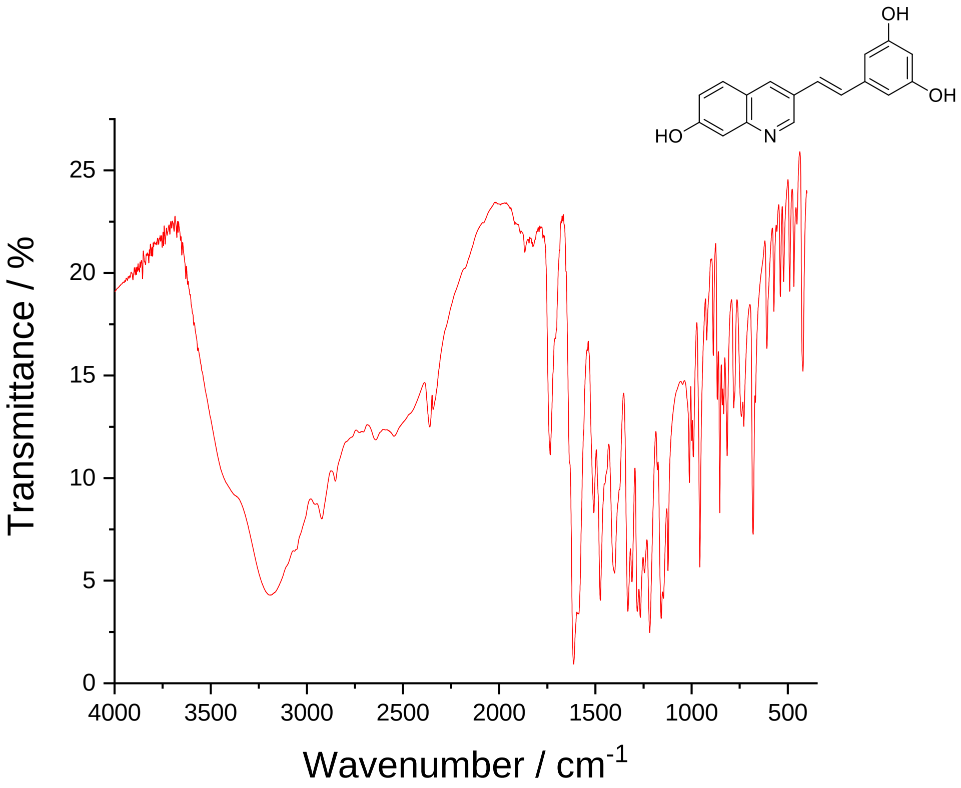
OH
HO
N
OH
Transmittance / %
Wavenumber / cm-1
0
5
10
15
20
25
4000
3500
3000
2500
2000
1500
1000
500

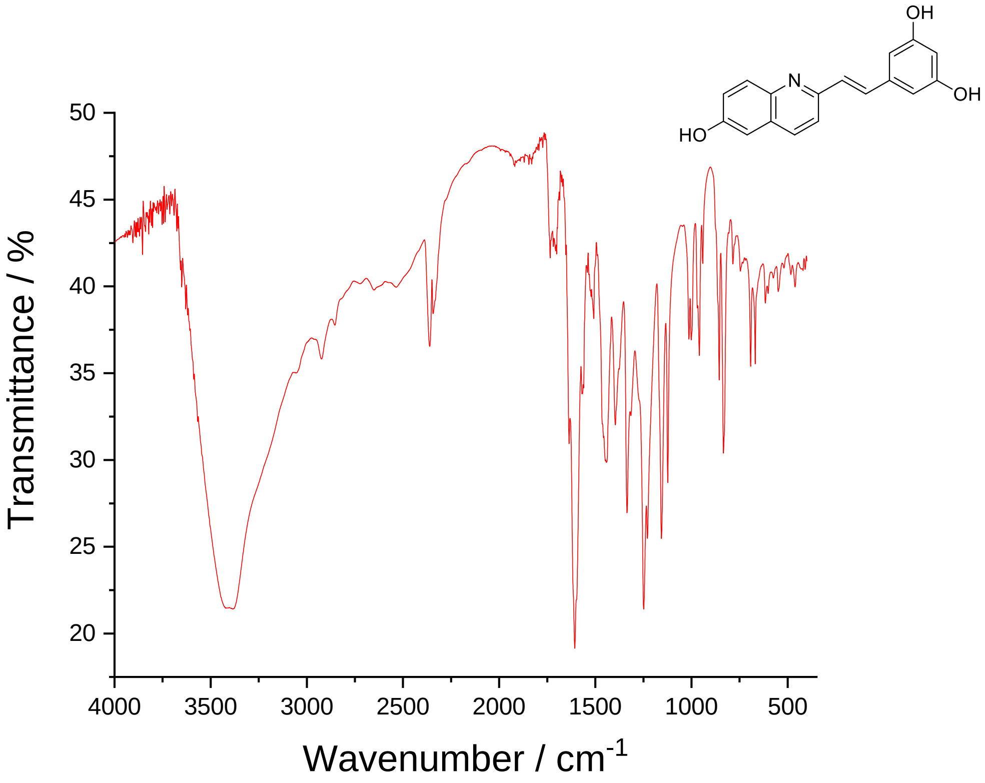
OH
N
HO
OH
Transmittance / %
Wavenumber / cm-1
20
25
30
35
40
45
50
4000
3500
3000
2500
2000
1500
1000
500

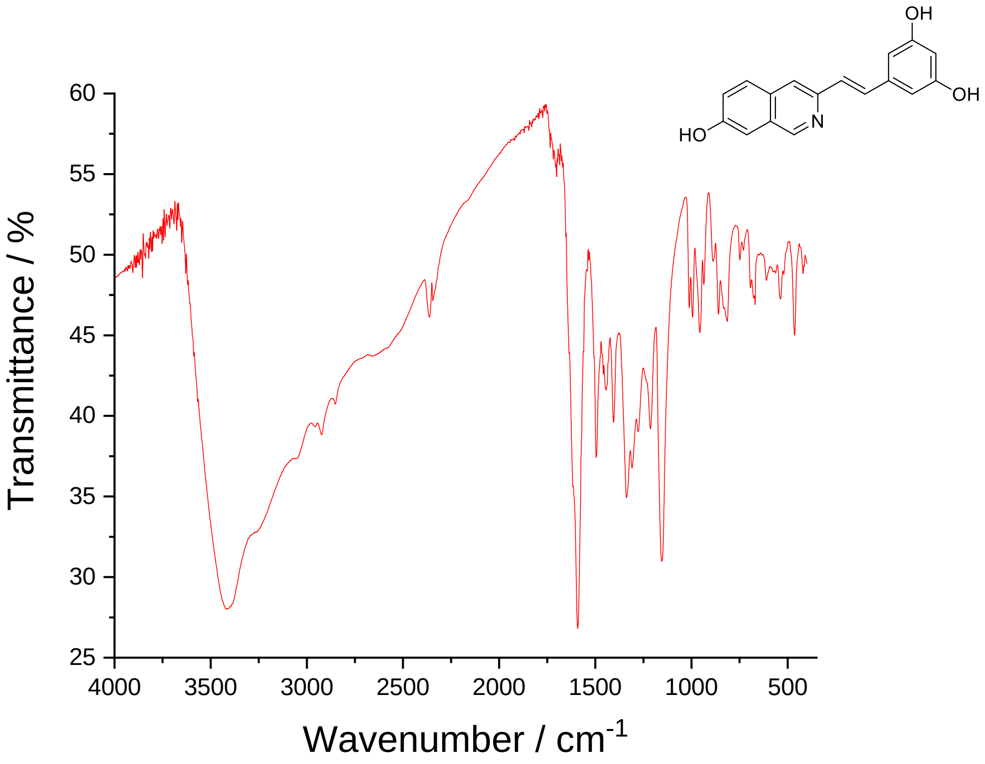
Transmittance / %
Wavenumber / cm-1
HO
N
OH
OH

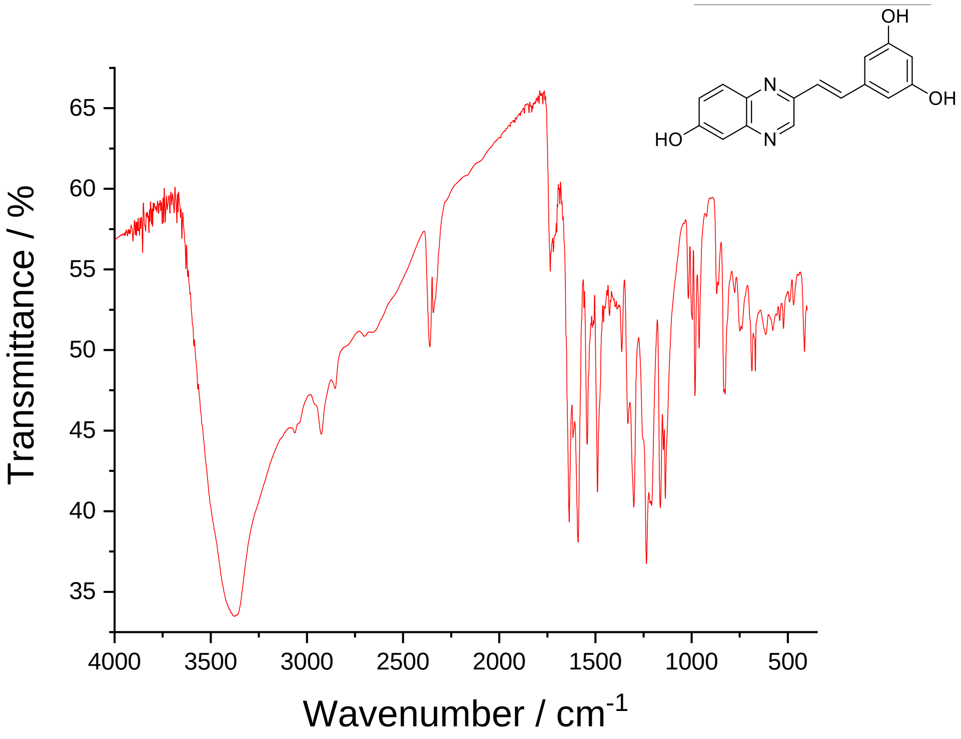
Transmittance / %
Wavenumber / cm-1
HO
N
N
OH
OH

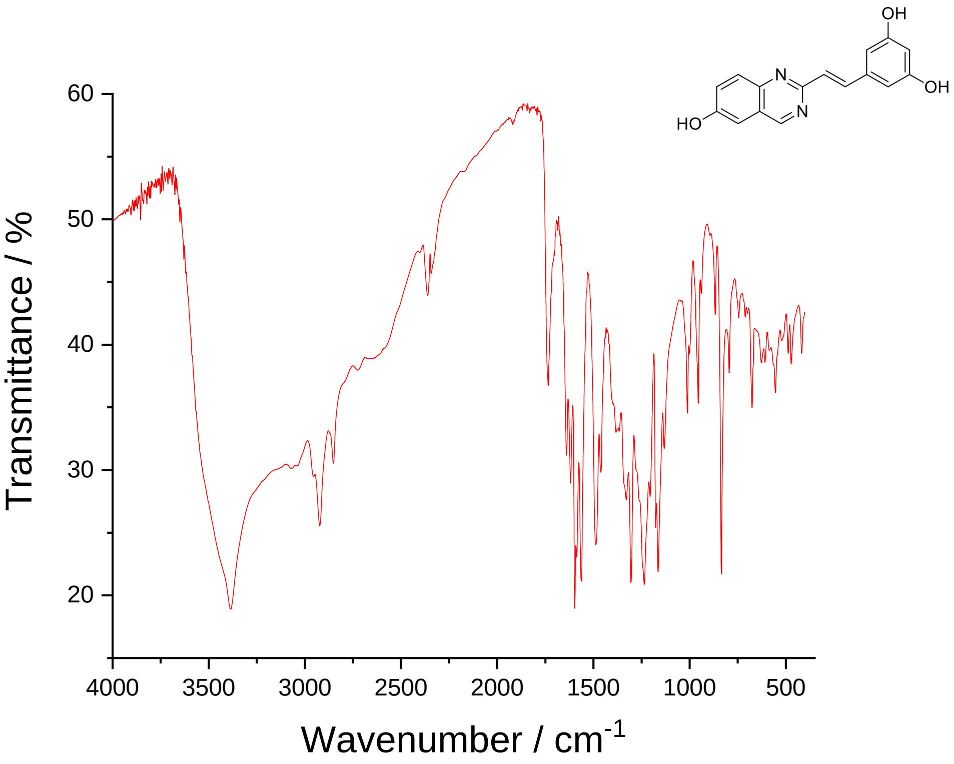

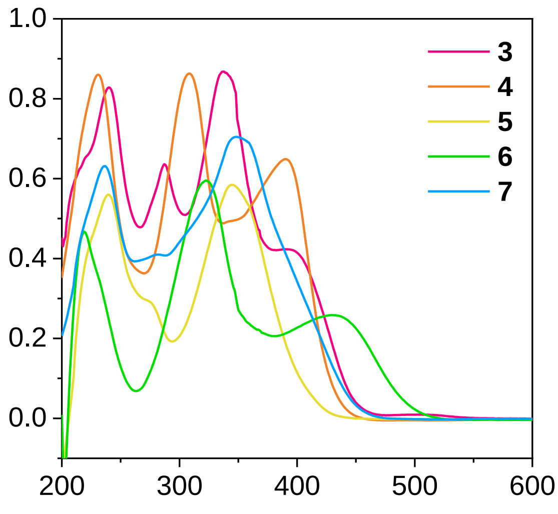

UV-Vis absorption spectra in MeOH of compounds **3**-**7**
